# Combination of visuo-tactile and visuo-vestibular correlations in illusory body ownership and self-motion sensations

**DOI:** 10.1371/journal.pone.0277080

**Published:** 2022-11-15

**Authors:** Nora Preuss Mattsson, Sara Coppi, Marie Chancel, H. Henrik Ehrsson

**Affiliations:** 1 Department of Neuroscience, Karolinska Institutet, Stockholm, Sweden; 2 University Grenoble Alpes, CNRS, LPNC, Grenoble, France; Anglia Ruskin University, UNITED KINGDOM

## Abstract

Previous studies have shown that illusory ownership over a mannequin’s body can be induced through synchronous visuo-tactile stimulation as well as through synchronous visuo-vestibular stimulation. The current study aimed to elucidate how three-way combinations of correlated visual, tactile and vestibular signals contribute to the senses of body ownership and self-motion. Visuo-tactile temporal congruence was manipulated by touching the mannequin’s body and the participant’s unseen real body on the trunk with a small object either synchronously or asynchronously. Visuo-vestibular temporal congruence was manipulated by synchronous or asynchronous presentation of a visual motion cue (the background rotating around the mannequin in one direction) and galvanic stimulation of the vestibular nerve generating a rotation sensation (in the same direction). The illusory experiences were quantified using a questionnaire; threat-evoked skin-conductance responses (SCRs) provided complementary indirect physiological evidence for the illusion. Ratings on the illusion questionnaire statement showed significant main effects of synchronous visuo-vestibular and synchronous visuo-tactile stimulations, suggesting that both of these pairs of bimodal correlations contribute to the ownership illusion. Interestingly, visuo-tactile synchrony dominated because synchronous visuo-tactile stimulation combined with asynchronous visuo-vestibular stimulation elicited a body ownership illusion of similar strength as when both bimodal combinations were synchronous. Moreover, both visuo-tactile and visuo-vestibular synchrony were associated with enhanced self-motion perception; self-motion sensations were even triggered when visuo-tactile synchrony was combined with visuo-vestibular asynchrony, suggesting that ownership enhanced the relevance of visual information as a self-motion cue. Finally, the SCR results suggest that synchronous stimulation of either modality pair led to a stronger illusion compared to the asynchronous conditions. Collectively, the results suggest that visuo-tactile temporal correlations have a stronger influence on body ownership than visuo-vestibular correlations and that ownership boosts self-motion perception. We present a Bayesian causal inference model that can explain how visuo-vestibular and visuo-tactile information are combined in multisensory own-body perception.

## Introduction

Previous research has shown that the perceptual experience of one’s own body is due to the dynamic integration of sensory signals from different sensory modalities [1–6]. For example, we receive tactile feedback when we touch our body, we can look at our body, we can listen to the steps of our feet when we walk, we can feel fatigue and shortness of breath if we walk too fast, and although less consciously than the previously mentioned examples, we feel the motion and orientation of our head and gravitational forces on our feet when we walk and move around. The vestibular system constitutes our ‘sense of balance’ and offers us a gravitational reference frame that allows us to navigate the world with our body. Furthermore, it helps us stay upright and stabilize our eye movements when we move our heads. However, most previous studies investigating the multisensory perceptual experience of one’s own body (body ownership) have used combinations of visual and somatosensory cues [2, 6–10], although interoceptive contributions have also spurred increasing interest [11–15]. However, recent evidence suggests that the vestibular system also plays an important role in the feeling of body ownership [16] through integration of visual and vestibular cues. This raises the question of how vestibular, visual, and somatosensory information together contribute to the feeling of body ownership, which speaks to the more general question of how multiple (more than two) sensory modalities are combined to create coherent multisensory representations of one’s own body.

Over twenty years ago, Botvinick and Cohen reported the ‘rubber hand illusion’ [2]. In the rubber hand illusion, participants have the illusion that a rubber hand is part of their own body. The rubber hand is placed in front of the participant, while the real hand is hidden behind a screen. Then, both hands are either synchronously or asynchronously stroked with a brush. During the synchronous stimulation condition, participants perceive that what they feel and see comes from the same source, which induces the illusion of owning a rubber hand [17, 18]. Ten years later, Petkova and Ehrsson [10] conducted a study using a plastic mannequin to induce a full-body ownership illusion. Participants wore a head-mounted display (HMD) watching a plastic mannequin from a first-person perspective (1PP) instead of their own body. Then, synchronous or asynchronous strokes using a small rod were applied to the real body and the mannequin’s body on corresponding sites on the abdomen. Similar to the rubber hand illusion, the participants’ visual and somatosensory information perceptually fuse, eliciting an illusion of the mannequin’s body being one’s own.

Previous studies that investigated the contributions of the vestibular system to body perception reported a modulating effect of vestibular stimulation on somatosensory perception [19–23], implicit representation of hand size and shape [23, 24], subjective ownership sensations [25] and proprioceptive drift [26] in the rubber hand illusion. A recent study investigated vestibular contributions to full-body ownership perception and observed that a full-body ownership illusion could also be induced using synchronous visuo-vestibular stimulation [16]. In a series of experiments, participants saw a mannequin lying on a bed from the first-person perspective (1PP) that passively and slightly rotated in an oscillating manner to one side. At the same time, they were exposed to galvanic vestibular stimulation (GVS), a noninvasive stimulation technique that induces a similar oscillating self-motion sensation to the side (for a review on GVS, see [27]). Visuo-vestibular stimulation could, hence, be either synchronous or asynchronous. In this previous study, we conducted a total of three experiments and compared a bimodal synchronous visuo-vestibular stimulation condition (“congruent”) to a condition where participants were looking at the mannequin moving without receiving GVS (“visual only”) and to a condition where participants looked at a stationary image of the mannequin while exposed to the GVS (“vestibular only”). We were able to show that synchronous visuo-vestibular stimulation was sufficient to induce an ownership illusion, and the asynchronous mode of stimulation served as a good control, significantly suppressing the illusion, in otherwise identical conditions.

The purpose of the present study was to combine visuo-tactile and visuo-vestibular stimulation in one experimental procedure to investigate how synchronous and asynchronous combinations of three different sensory modalities contribute to the perception of a body as one’s own. This is an important question because own-body perception under natural conditions involves correlated sensory feedback from many sensory modalities simultaneously, and the vast majority of previous studies on the rubber hand illusion and full-body illusions have only manipulated the spatiotemporal correspondences of two modalities at a time. We registered subjective illusory body ownership and self-motion using questionnaire ratings and skin conductance response (SCR) elicited by physical threats directed to the mannequin, which served as indirect physiological evidence for the body ownership illusion (indexing autonomic arousal and emotional defense reactions; [10, 28, 29]). We expected that both synchronous visuo-tactile and synchronous visuo-vestibular information would contribute to the ownership illusion in line with earlier work [10, 30]. More critically, we wanted to assess which of the two pairs of sensory correlations would dominate and if the illusion could still be elicited if one of the two pairs of bimodal stimulation was asynchronous and the other synchronous. Furthermore, based on the results of a previously conducted study [30], we expected that both synchronous visuo-tactile and synchronous visuo-vestibular stimulation would lead to stronger self-motion perception, and again, we were interested in characterizing the relative contribution of the visuo-vestibular and visuo-tactile correlations. Finally, to better understand how the visuo-vestibular and visuo-tactile information was combined in the current paradigm, we adapted a Bayesian causal inference model of body ownership [17, 18] to the special case of how two pairs of sensory modalities lead to a coherent perceptual experience of the body in space based on probabilistic computational principles from the theoretical framework of multisensory integration (see further below). The key difference from typical causal inference models, which aim to explain how two sensory inputs about a single event are combined, is that the current model describes how two pairs of sensory signals from three modalities are combined to give rise to body ownership. The model has a hierarchical structure where at the first level, visual and tactile signals and visual and vestibular signals are combined or segregated according to causal inference principles as two separate processes; the estimates from both these processes are then averaged according to their relative reliability in a “global” causal inference process at a second level, leading to the multisensory percept of the whole body in view as one’s own.

## Methods

### Participants

All subjects were naïve and healthy participants recruited through online and physical advertisements (they were not psychology undergraduates who did the study for a course credit). A total of 85 healthy participants with normal or corrected-to-normal vision were recruited. Five were excluded because they did not complete the experiment, leaving us with 80 participants who were used in the analyses (37 male, mean age = 25, SD = 4.42, 76 right-handed, 1 ambidextrous, 3 left-handed according to the Edinburgh Handedness inventory by [31]. Of those participants, 30 completed only the questionnaire experiment, and the remaining 50 conducted both the questionnaires and the threat-evoked skin conductance responses. The sample sizes were determined before the study started and were larger than previous studies on full-body illusion ownership, which included 20–48 participants. Since we were interested in comparing the relative contributions of visuo-vestibular and visuo-tactile synchrony/asynchrony, we reasoned that such differences might be smaller than the basic effect of synchronous versus asynchronous visuo-tactile conditions [10, 16, 32] and that a larger sample size would also improve the conclusiveness of the Bayesian analyses that we wanted to include to follow-up on the effects of potential similarly strong illusions in two conditions (see further below). For the SCR analyses, we recruited 52 participants but analyzed data from only 50 participants, as two participants did not finish the procedure (23 male, mean age = 24.52, SD = 3.82). One of the participants included in the SCR analysis was left-handed, as indicated by the Edinburgh Handedness Questionnaire [31], and two left-handers and one ambidextrous people were included in the questionnaire data analysis. A separate group of twenty-five participants was tested in a pilot experiment (see ‘Supporting Information–Section I’ in S1 File), and we confirmed that the new kind of threat stimulus used in the SCR procedure worked as expected (17 male, mean age = 28.08, SD = 6.27, 23 right-handed, 1 left-handed, 1 ambidextrous).

The Swedish Ethics Review Authority approved the experimental procedure. All participants gave written informed consent.

### Galvanic vestibular stimulation

Participants were exposed to GVS using a DC stimulator (neuroCon GmbH, Illmenau, Germany). Electrode sponges (9 cm^2^) were soaked in sodium chloride solution (B. Braun Melsungen AG, Germany) and then, together with the rubber electrodes (9 cm^2^), attached behind participants’ ears. The anode was placed behind the left ear, and the cathode was placed behind the right ear, as it is known that GVS causes sway to the anodal side in standing people [33]. The stimulation protocol was based on a previous study [16], and the strength of the stimulation was adjusted individually (range 0.7 mA to 2 mA), as the participants differed in vestibular and pain sensitivity (for an overview, see [16]). We started by applying a sinusoidal current pulse (1 mA, frequency 1 Hz), which elicited a brief feeling of combined translation movement to the left and clockwise rotation in the roll plane. Next, participants underwent a calibration measurement, where they had to verbally describe their perceived motion sensation and indicate the sensation with their hand. The experimenter followed a staircase procedure increasing the intensity of the pulses until the participants reported feeling a movement. The mean stimulation intensity was 1.26 mA (SD = ± 0.151 mA) in the questionnaire experiment and 1.384 mA (SD = ± 0.122 mA) in the SCR experiment. The participants were instructed that the vestibular stimulation should lead to a clear sense of rotation but not be painful or uncomfortable. Stimulation was increased or decreased in steps of 0.5 mA depending on participant verbal feedback about motion sensation to achieve a suitable stimulation strength adjusted for each individual. Each GVS stimulation lasted 1 second.

### Stimuli and apparatus

**Fig 1:**
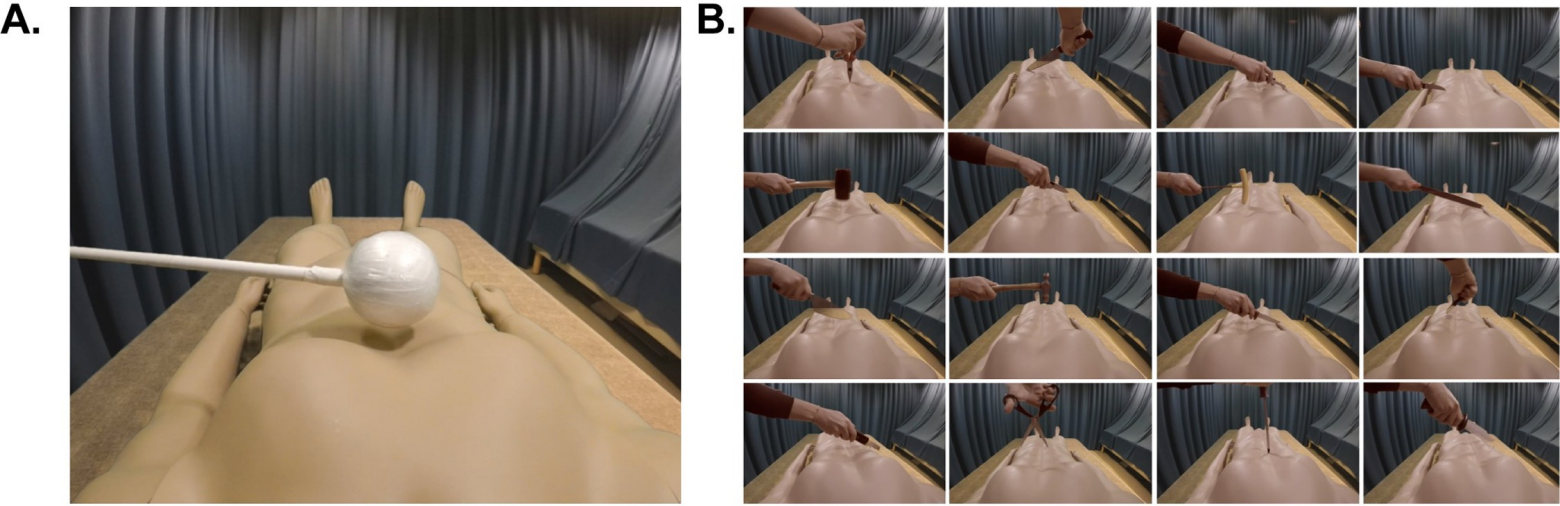
Visual material for the questionnaire and the SCR experiments. (**A**). The participant lay on a bed with his or her head tilted forward, wearing a head–mounted display (HMD). In the HMD, the participants saw a mannequin lying on a bed from a first–person perspective, and they were exposed to temporally synchronous or asynchronous visuo–tactile stimulation (0.5 Hz) using a white styrofoam ball attached to a wooden stick. At the same time, the visual background made a brief rotation movement (oscillated 3°/sec) around the mannequin once every six seconds to induce an illusory clockwise motion sensation in the roll plane in the participants. These visual oscillations were presented synchronously or asynchronously with the electrical stimulation of the vestibular nerve (GVS), which elicited a brief sensation of clockwise rotation in the roll plane and translation to the left. (**B**). Sixteen different physical threat stimuli were used to register the participants’ threat–evoked skin conductance responses.

In the HMD, the participants saw prerecorded videos of a male mannequin body lying on a bed from the natural point of view (1PP) (Fig 1A). All participants, irrespective of sex, saw the same mannequin, as previous studies did not report a significant influence of the mannequin’s sex on the ownership illusion in males and females [10, 34]. To create a three-dimensional (3D) visual scene, videos were recorded using two identical cameras placed side-by-side (Go-Pro Hero 5, resolution 1920x1080) and a green screen setup. The video material was edited using Final Cut Pro X (Apple Final Cut Pro X License and Download [Electronic Resource]). Based on a previous study [16], a visual motion sensation was induced using a 3°/sec oscillating rotation of the background stimulus (starting counterclockwise) to match the GVS-induced motion sensation. This visual rotation induces a ‘hammock-like’ self-motion sensation to the left when presented to participants in a wide visual field. GVS and visually induced motion were synchronized using a customized program that triggered the start of the GVS pulses. The trigger was sent when the visual motion started in the synchronous condition and with a 2-sec delay in the asynchronous condition. The frequency of the GVS pulses was based on a previous study [16] and occurred once every 7 seconds. Visuo-tactile stimulation was applied to the abdomen of the mannequin and the abdomen of the participant using a thin wooden stick (1 m) with a white styrofoam ball attached to the top (see Fig 1A). In the videos, the mannequin was naked and lay supine on a bed in the experimental room. The white ball had a diameter of 8 cm. The strokes applied on the abdomen were approximately 15 cm long and always started just below the chest and moved toward the center of the abdomen. The duration of each stroke was 1 sec. To induce asynchronicity between visual and tactile stimulations, we added a one-second delay to the tactile stimulation compared to the visual stimulus. The frequency of strokes was approximately one every two seconds (0.5 Hz) in line with an earlier full-body illusion study [35]. The seen and felt strokes were always applied on the corresponding parts of the abdomens of the mannequin and the participant’s (unseen) real body; thus, in the synchronous and asynchronous visuo-tactile conditions, it was only the temporal congruence that was being manipulated. The experimenter received audio cues through headphones to ensure proper manual delivery of the tactile stimulation to the participant’s real body depending on the condition.

For the SCR analysis, we presented a ‘threat’ toward the mannequin’s abdomen in the second half of the video [16]. To reduce attenuation effects [16], we used a total of 16 different types of threats involving different knives and sharp and blunt tools; hence, 16 different videos were prepared with lengths between 59 and 67 seconds (Fig 1B), according to similar full-body illusion experiments that used a period of 60 seconds per condition [36, 37] or between 40 and 80 seconds [7]. The threat episodes lasted for 1 sec and were presented randomly between second 39 and second 64 so that the participant could not anticipate their precise occurrence yet had time first to develop the bodily illusion. The effectiveness of this particular set of threat stimuli as objective psychophysiological evidence of the full-body illusion was confirmed in a separate pilot experiment (see ‘Supporting Information—Section I’ in S1 File).

### Procedure

#### Questionnaire experiment

A total of 80 participants were recruited and included in the questionnaire experiment. Participants were lying on a bed with their heads tilted forward approximately by 45 degrees. The head position was stabilized with a pillow that was taped to the bed. Video material was presented using an HMD worn by the participants (Oculus Rift 2, https://www.oculus.com), through which they could see the mannequin and the surrounding scene. The experiment consisted of four different conditions that were presented in randomized and counterbalanced order: visuo-vestibular (^VV^) synchrony (S) and visuo-tactile (^VT^) synchrony, i.e., the S^VV^S^VT^ condition; visual-vestibular asynchrony (A) and visuo-tactile synchrony, i.e., the A^VV^S^VT^ condition; visual-vestibular synchrony and visuo-tactile asynchrony, i.e., the S^VV^A^VT^ condition; and visuo-vestibular asynchrony and visuo-tactile asynchrony, i.e., the A^VV^A^VT^ condition (see Table 1). Each condition lasted for 3 minutes, and participants had to rate six questionnaire statements on a seven-point Likert scale from -3 (fully disagree) to +3 (fully agree) after each condition to measure the subjective experience of illusory ownership (see Table 2). Statement 1 (S1: ‘it felt as if I was looking at my body’) of the questionnaire is the most important in the current study since it catches the subjective experience of owning a fake body regardless of the stimulated sensory modality. Statements 2 and 3 (S2: ‘the body motion I saw was the motion I felt’; S3: ‘it seems as though the touch I felt was caused by the white ball’) relate to visuo-vestibular and visuo-tactile binding that occurs because of congruence within a specific pair of sensory modalities. We administered a 7-point Likert scale (from -3 to +3) that is commonly used to investigate the subjective experience in rubber-hand illusion experiments [2, 25, 38] as well as in full-body ownership experiments [16, 30], where subjects are asked to either affirm or deny the ownership statements. In addition, we used a 10-point visual analog scale (0: “not at all”; 10: “very much”) (see Table 2) to quantify perceived self-motion. When filling out the questionnaires, the participants removed the HMD.

**Table 1:** 2x2 factorial design.

		*VISUO-VESTIBULAR STIMULATION*	
		*Synchronous*	*Asynchronous*
***VISUO-TACTILE STIMULATION***	*Synchronous*	**S** ^**VV**^ **S** ^**VT**^	**A** ^**VV**^ **S** ^**VT**^
	*Asynchronous*	**S** ^**VV**^ **A** ^**VT**^	**A** ^**VV**^ **A** ^**VT**^

**Table 2:** Illusory ownership questionnaire. Statements S1–S6 were measured using a 7–point Likert scale, while S7 was measured using a 10–point visual analog scale.

*Statement*	*During the experiment:*	*Type*
*S1*	…it felt as I was looking at my body.	Body ownership
*S2*	…the body motion I saw was the motion I felt.	Visuo-vestibular binding
*S3*	…it seems as though the touch I felt was caused by the white ball.	Visuo-tactile binding
*S4*	…it felt as if I had two bodies.	Illusion control
*S5*	…I felt as if my body was turning ‘plastic’.	Illusion control
*S6*	…I felt dizzy.	Dizziness control
*S7*	…how much motion did you feel? Please indicate.	Self-motion

#### SCR experiment

For the 50 participants who also took part in the threat-evoked SCR (recorded via a Biopac System MP150, Goleta, U.S.A.), we made a few minor adjustments in the procedures; all other procedures were identical to what is described above for the questionnaire experiment. We used a total of 16 shorter trials (between 59 and 67 seconds) so that we could collect data from multiple threat events. The experimental conditions S^VV^S^VT^, S^VV^A^VT^, A^VV^S^VT^ and A^VV^A^VT^ were presented in a pseudorandomized order so that every condition was presented equally often at the first, second, third or fourth position within each block of four trials. There were four different randomization possibilities: S^VV^S^VT^-A^VV^A^VT^-A^VV^S^VT^-S^VV^A^VT^; A^VV^S^VT^-S^VV^S^VT^-S^VV^A^VT^-A^VV^A^VT^; S^VV^A^VT^-A^VV^S^VT^-A^VV^A^VT^-S^VV^S^VT^; and A^VV^A^VT^-S^VV^A^VT^-S^VV^S^VT^-A^VV^S^VT^. Every participant was assigned to one of these randomization orders, and each order was repeated a total of four times. The whole procedure took approximately 20 minutes, and there was no break between the first 12 trials; during these trials, only SCR data were collected. Questionnaire data were collected once for each condition after the last four trials (trials 13–16) (participants took off the HMD to do so). Regarding the threat-evoked SCR, we had 16 short videos with 16 different knife threats that were presented to the participants at an unpredictable time point in the second half of each trial in a fully randomized order.

#### Pilot experiment

Prior to the main study described above, we conducted a pilot experiment to validate the new experimental procedure with threats to the mannequin performed with 16 kinds of knives, hammers, paper cutters, screwdrivers, etc. (see Fig 1B). This approach was inspired by SCR studies involving pictures of emotional stimuli (e.g., spiders or snakes), where it is a common procedure to use different pictures to reduce attenuation effects. In previous studies, we only used a single threatening object, typically a knife [10, 34, 39, 40], and we reasoned that varying the threat stimulus could potentially reduce attenuation to repeated presentations that are otherwise present in the data [16]. In the pilot experiment, we only tested the visuo-tactile synchronous and asynchronous conditions and measured the effect on subjective experience as well as on the SCR (‘Supporting Information—Section I, S1 and S2 Figs’ in S1 File). The full procedure is described in the ‘Supporting Information–Section I’ in S1 File.

### Analysis

All analyses were performed using Rstudio [41], and the alpha level was set to 5%. Mixed effect models were calculated using the clmm2 function of the “ordinal” package (version 2019.12–10) [42] and rlmer of the “robustlmm” package [43] in R. In addition to p values, we report Bayes factors (BFs) for planned comparisons. BF indicates whether the data support the alternative Hypothesis H_1_ or the null Hypothesis H_0_. For example, a BF_10_ of 5 would indicate that the observed data are 5 times more likely to have happened under H_1_ than under H_0_ [44]. A BF_10_ of 1 indicates neither evidence for H_1_ nor H_0_. Usually, a BF_10_ of 1–3 is termed ‘anecdotal’ evidence, and a BF_10_ larger than 3 is termed ‘moderate’ evidence [45]. BFs for planned comparisons were calculated using the “BayesFactor” package for R (version 0.9.12–4.3) [46], and the prior of the effect size was set to its default value of sqrt(2)/2. Note that BF_10_ in the current paper refers to evidence in favor of the alternative hypothesis, so moderate evidence in favor of the null hypothesis would correspond to a BF_10_ < 0.33. In addition, for planned comparisons, we report the results of either a Wilcoxon signed-rank test or a t test depending on the distribution of the data. The normality of the data was checked by the Shapiro-Wilk test (a deviation from normality is shown by a Shapiro-Wilk p value < .05). We also reported the matched pairs rank biserial correlation (“*r*_*C*_”, which stands for “matched-pairs rank-biserial correlation”) as the effect size for the Wilcoxon signed-rank test [47, 48] and the Cohen’s coefficient “*d*_*z*_” as the effect size for the t test [49]. Graphs were made by the use of the package “ggplot2” for R [50].

#### Questionnaire data analysis

The questionnaire ratings were measured using a seven-point Likert scale from -3 to +3 (strongly disagree to strongly agree) (for the bodily illusion, S1-S6) and a 10-point visual analog scale (for the self-motion illusion, S7). Data analysis was performed using a cumulative link mixed model for each ordinal questionnaire statement. Maximum likelihood estimates of the parameters were estimated using the adaptive Gauss-Hermite quadrature method (10 nodes) [42]. Visuo-tactile and visuo-vestibular stimulation (synchronous, asynchronous) were entered as fixed effects, and participant ID was entered as a random effect. Likelihood ratio tests were performed to test the fixed effect while controlling for the remaining variables. Questionnaire data were analyzed in a single model as the predictor ‘participant group’ did not have a significant influence on statement S1 (the most important experimental statement in the current study as it relates to the overall full-body illusion experience and not illusory sensations related to the specific pair of bimodal stimuli that are congruent or incongruent as in S2 and S3) (χ^2^(1) = 0.938, *p* = 0.333). The model regarded both those participants who only took part in the questionnaire part and those who took part in both the questionnaire and SCR parts.

As described above, we used the questionnaire data from all 80 participants who underwent this procedure in a single analysis to maximize statistical power since we were interested in comparing the relative contributions of visuo-vestibular and visuo-tactile congruence and reasoned that such differences may be smaller than the basic effect of synchronous versus asynchronous visuo-tactile conditions [10] and that a larger sample size would also improve the conclusiveness of the Bayesian analyses (see further below). We run a post hoc 1-tail power analysis on the questionnaire experiment based on the “*r*_*C*_” effect sizes [48]. We used G*Power [51], and as a statistical test, we selected “Correlation: Point biserial model”. In statement one (S1), the relative contribution of the visuo-vestibular correlation to body ownership is shown by the comparisons S^VV^S^VT^ versus A^VV^S^VT^ and S^VV^A^VT^ versus A^VV^A^VT^. For the first comparison, the effect size was *r*_*C*_ = -0.037 with a power of 7.5%. In the second comparison, the effect size was *r*_*C*_ = 0.562 with a power of 97%. The relative contribution of the visuo-tactile correlation to body ownership is shown by the comparisons S^VV^S^VT^ versus S^VV^A^VT^ and A^VV^S^VT^ versus A^VV^A^VT^. The effect size for the first comparison was *r*_*C*_ = 0.476, and its power was 89%; the effect size for the second comparison was *r*_*C*_ = 0.947, and its power was 100%.

Wilcoxon signed-rank tests were performed for planned comparisons. We expected that the ownership ratings in S^VV^S^VT^ should be higher than those in A^VV^A^VT^, A^VV^S^VT^ and S^VV^A^VT^. Furthermore, we planned to compare the ratings in A^VV^S^VT^ and S^VV^A^VT^. Descriptive statistics and post hoc questionnaire analyses are provided in ‘Supporting Information–Section II, S3 and S4 Tables’ in S1 File.

#### SCR data analysis

We analyzed the SCR magnitude induced by means of physical threats to the mannequin’s body in the SCR experiment. SCR magnitude has been used in many previous full-body illusion studies [10, 39, 52] and is a straightforward approach to obtain a measure of total SCR response to threat stimuli. The average response across multiple trials, including only those where the SCR was elicited (i.e., > .01 mmho), is called ‘amplitude’, whereas the average response that also includes trials with null responses (i.e., ≥ 0 mmho) is called ‘magnitude’. Sometimes magnitude and amplitude are distinguished because an effect of magnitude can be driven by a higher response frequency and not necessarily a stronger response [53]. However, the proportion of null responses (SCR < 0.01 mmho) in the current study was very low (0.02%, i.e., only 16 trials out of 800 had a raw value < 0.01 mmho); thus, we decided to analyze the magnitude responses. This is also in line with several of our previous studies as described above [16, 54].

SCR magnitude data were analyzed using a linear mixed-effects model, which also allowed us to consider order effects. SCR magnitude was set as the dependent variable and predicted by repetition, visuo-tactile congruence, visuo-vestibular congruence and interaction between the latter two. We included a random intercept per subject to account for subject variability. Given the slightly left-skewed data (skeweness = -0.1), we ran a robust linear mixed-effects model [43]. All SCR measurements were range-corrected to account for interindividual differences and standardize the measurements to the same scale. To this end, for every participant, each response was normalized by dividing it by the strongest value of each participant’s responses (see also [16]). Planned comparisons were analyzed post hoc using t tests since the differences between conditions were normally distributed. Hypotheses were tested one-sided, as we had the strong expectation that synchronous stimulation should result in stronger SCR magnitude than the asynchronous condition based on previous literature [10, 16, 55, 56].

To verify the robustness of the results from the above analysis strategy, we conducted three extra analyses where we (i) analyzed the SCR data without normalization; (ii) analyzed the normalized SCR data but excluded the two participants that had very weak SCR amplitudes (“null responders”); and (iii) analyzed the nonnormalized SCR data excluding the null responders (as in [10]). These post hoc analyses are included purely for descriptive purposes, and we did not expect to find any significant differences between the different analytical approaches. See the results of these analyses in the ‘Supporting Information–Section III’ in S1 File.

#### Bayesian causal inference model

After the experiments had been conducted and the data were analyzed, we developed a Bayesian causal inference model to explain how our findings can be understood within a probabilistic computational framework of multisensory integration [17, 18]. For clarity, the details of the model are described in the results section, as well as a post hoc correlation analysis of the questionnaire data that was inspired by the model.

## Results

### Questionnaire data

Concerning ownership statement S1, the cumulative link mixed model revealed that both predictors, visuo-tactile and visuo-vestibular congruence, contributed to the ownership illusion. There was a significant effect for the predictor visuo-tactile congruence, χ^2^(1) = 30.489, *p* < 0.001, and a near-significant effect for the predictor visuo-vestibular congruence in the hypothesized direction, χ^2^(1) = 3.384, *p* = 0.066 (note that a two-tailed test was used). There was, however, no significant interaction effect, χ^2^(1) = 0.884, *p* = 0.347. Planned comparison for S1 showed a greater score in the fully synchronous condition S^VV^S^VT^ than the fully asynchronous condition A^VV^A^VT^ (*V* = 1227, *p* < 0.001, BF_10_ > 100, *r*_*C*_ = 0.715) and a greater score than the condition when only the visuo-tactile stimulation was asynchronous S^VV^A^VT^ (*V* = 1217, *p* < 0.001, BF_10_ > 100, *r*_*C*_ = 0.525). However, there was no significant difference when comparing the fully synchronous condition (S^VV^S^VT^) to the condition with asynchronous visuo-vestibular stimulation and synchronous visuo-tactile, even though the Bayesian analysis indicated only ‘anecdotal’ evidence in favor of the null hypothesis (A^VV^S^VT^, *V* = 482, *p* = 0.152, BF_10_ = 0.338, *r*_*C*_ = 0.176). Interestingly, there was a significant difference when comparing the effect of S^VV^A^VT^ with the effect of A^VV^S^VT^ (*V* = 357, *p* = 0.005, BF_10_ = 6.124, *r*_*C*_ = -0.44). Both these conditions produced affirmative mean ownership ratings (over zero) (S^VV^A^VT^: M = 0.462, SD = ± 0.1896, Mdn = 1; A^VV^S^VT^: M = 0.938, SD = ± 1.641, Mdn = 1), but the illusion experienced was significantly less vivid when the visuo-tactile stimulation was asynchronous, indicating that violating the visuo-tactile temporal congruence rule had a more disrupting effect on the full-body illusion than visuo-vestibular temporal incongruence.

The analysis of statement S2 concerning perceived visuo-vestibular binding revealed that both predictors significantly affected this aspect of the multisensory illusion. There was a significant effect of visuo-vestibular congruence, χ^2^(1) = 14.795, *p* < 0.001, as well as a significant effect of visuo-tactile congruence, χ^2^(1) = 44.684, *p* < 0.001, but no interaction effect, χ^2^(1) = 0.4, *p* < 0.527. Planned comparison for S2 showed that scores in the fully synchronous condition S^VV^S^VT^ were significantly greater than the scores in the fully asynchronous condition A^VV^A^VT^ (*V* = 2022.5, *p* < 0.001, BF_10_ > 100, *r*_*C*_ = 0.886), significantly greater than the condition when only the visuo-tactile stimulation was asynchronous (S^VV^A^VT^, *V* = 1125, *p* < 0.001, BF_10_ > 100. *r*_*C*_ = 0.765), and significantly greater than the condition when only the visuo-vestibular stimulation was asynchronous (A^VV^S^VT^, *V* = 899.5, *p* = 0.002, BF_10_ = 30.803, *r*_*C*_ = 0.469). However, there was no significant difference between S^VV^A^VT^ and A^VV^S^VT^ (*V* = 724.5, *p* = 0.076, BF_10_ = 0.615, *r*_*C*_ = -0.258).

The analysis of questionnaire statement S3, which concerned the referral of touch statements, revealed that only synchronous visuo-tactile stimulation had a significant effect, χ^2^(1) = 121.391, *p* < 0.001, which is expected. Planned post hoc comparisons revealed that in condition S^VV^S^VT^, scores were higher compared to S^VV^A^VT^ (*V* = 1464, *p* < 0.001, BF_10_ > 100, *r*_*C*_ = 0.972), and scores in A^VV^S^VT^ were higher than in condition A^VV^A^VT^ (*V* = 1829, *p* < 0.001, BF_10_ > 100, *r*_*C*_ = 0.873).

The analysis of control statement S4 revealed no significant effects (*p* > 0.1). The analysis of control statement S5 revealed an effect of visuo-tactile stimulation, χ^2^(1) = 11.007, *p* = 0.001 on questionnaire ratings. However, all control ratings were negative (i.e., < 0), meaning that most participants rejected these “made-up” experiences as expected; therefore, we will not interpret this result further. Statement S6 captured feelings of dizziness that might arise as a consequence of wearing the HMD and receiving the GVS stimulation, but no significant differences were observed across conditions (*p* > 0.1).

The results of S7 revealed that both visuo-tactile congruence and visuo-vestibular congruence significantly boosted the perception of self-motion (χ^2^(1) = 14.302, *p* < 0.001, χ^2^(1) = 12.9, *p* < 0.001; Fig 3). However, no interaction was found (χ^2^(1) = 0.092 *p* = 0.762). Planned comparison for S7 showed that scores in the S^VV^S^VT^ condition were higher than the scores in the A^VV^A^VT^ condition (*V* = 1641.5, *p* < 0.001, BF_10_ > 100, *r*_*C*_ = 0.681), higher than those scores in the S^VV^A^VT^ condition (*V* = 1315.5, *p* < 0.001, BF_10_ = 91.613), and higher than the scores in the A^VV^S^VT^ condition (*V* = 1174.5, *p* = 0.001, BF_10_ = 41.136, *r*_*C*_ = 0.472). However, no significant difference was found between S^VV^A^VT^ and A^VV^S^VT^ (*V* = 833.5, *p* = 0.958, BF_10_ = 0.127, *r*_*C*_ = 0.008). Thus, both visuo-vestibular and visuo-tactile synchrony led to stronger self-motion sensations.

**Fig 2:**
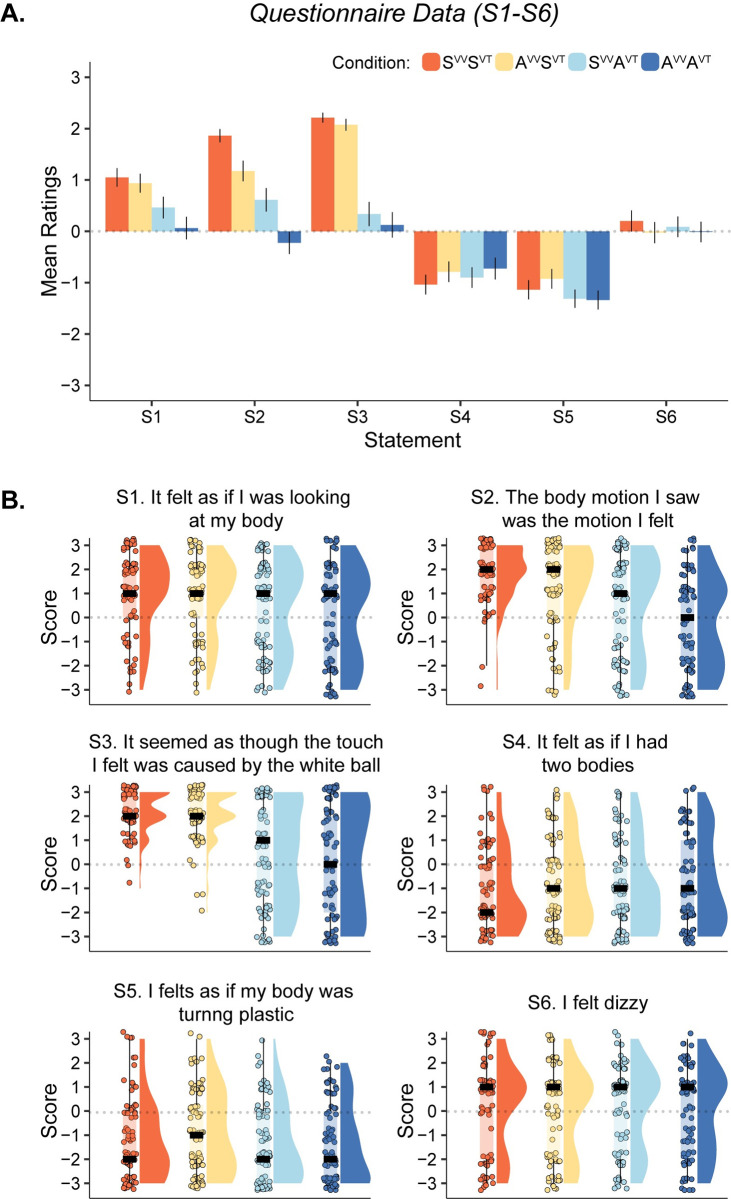
Plots for questionnaire results for statements S1–S6 (N = 80). (**A**). Means and standard errors of the mean are shown for all statements for illustrative purposes. (**B**). Raincloud plots show individual data points, medians, and distributions. Note: paired comparisons, descriptive statistics and post hoc comparisons are shown in S3 Fig, S3 and S4 Tables of the ‘Supporting Information–Section II’ in S1 File, respectively.

**Fig 3:**
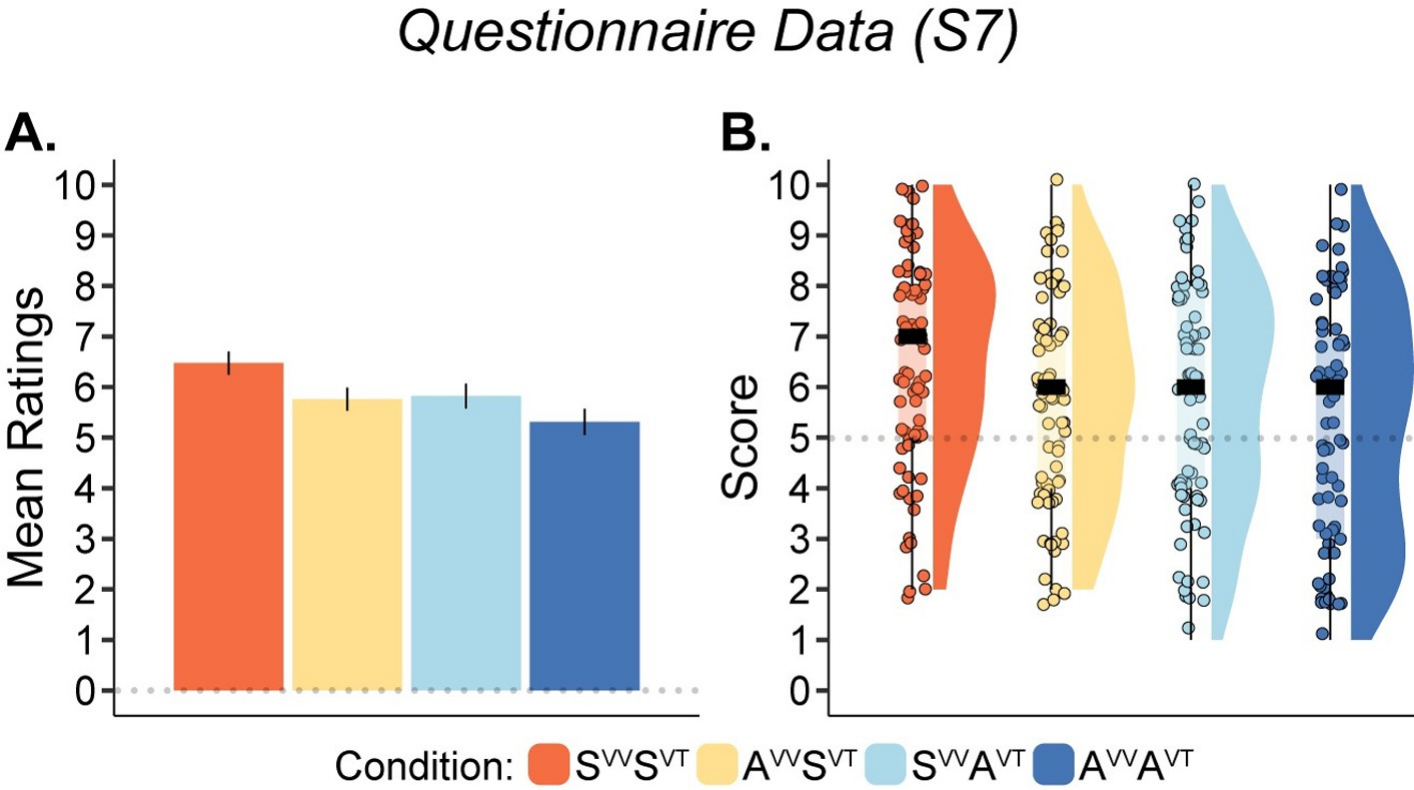
Plots for questionnaire results for statement S7 (N = 80) (“How much motion did you feel? Please indicate”). (**A**). Barplot shows the means and standard error of the mean for illustrative purposes. (**B**). Raincloud plots show individual data points, medians, and distributions. Note: paired comparisons, descriptive statistics and post hoc comparisons are shown in S3 Fig, S3 and S4 Tables of the ‘Supporting Information–Section II’ in S1 File, respectively.

The results of both experiments combined are illustrated in Figs 2 and 3. The results for each paired comparison for all statements as well as descriptive statistics are presented in the ‘Supporting Information–Section II, S3 Fig, S3 and S4 Tables’ in S1 File.

### SCR results

The robust linear mixed-effects model analysis of SCR magnitude in the SCR experiment revealed a main effect of visuo-tactile congruence (*β* = 0.097, *t* = 3.919, *p* < 0.001), showing that synchronous visuo-tactile stimulation resulted in significantly higher SCR than asynchronous visuo-tactile stimulation. The main effect for visuo-vestibular congruence did not reach significance (*β* = 0.035, *t* = 1.395, *p* = 0.163); in addition, there was no significant interaction between visuo-tactile and visuo-vestibular congruence (*β* = -0.068, *t* = -1.929, *p* = 0.054). Furthermore, there was an effect of order, showing the strongest response for the first knife threat and a decrease over time (*β* = -0.004, *t* = -1.967, *p* = 0.049).

**Fig 4:**
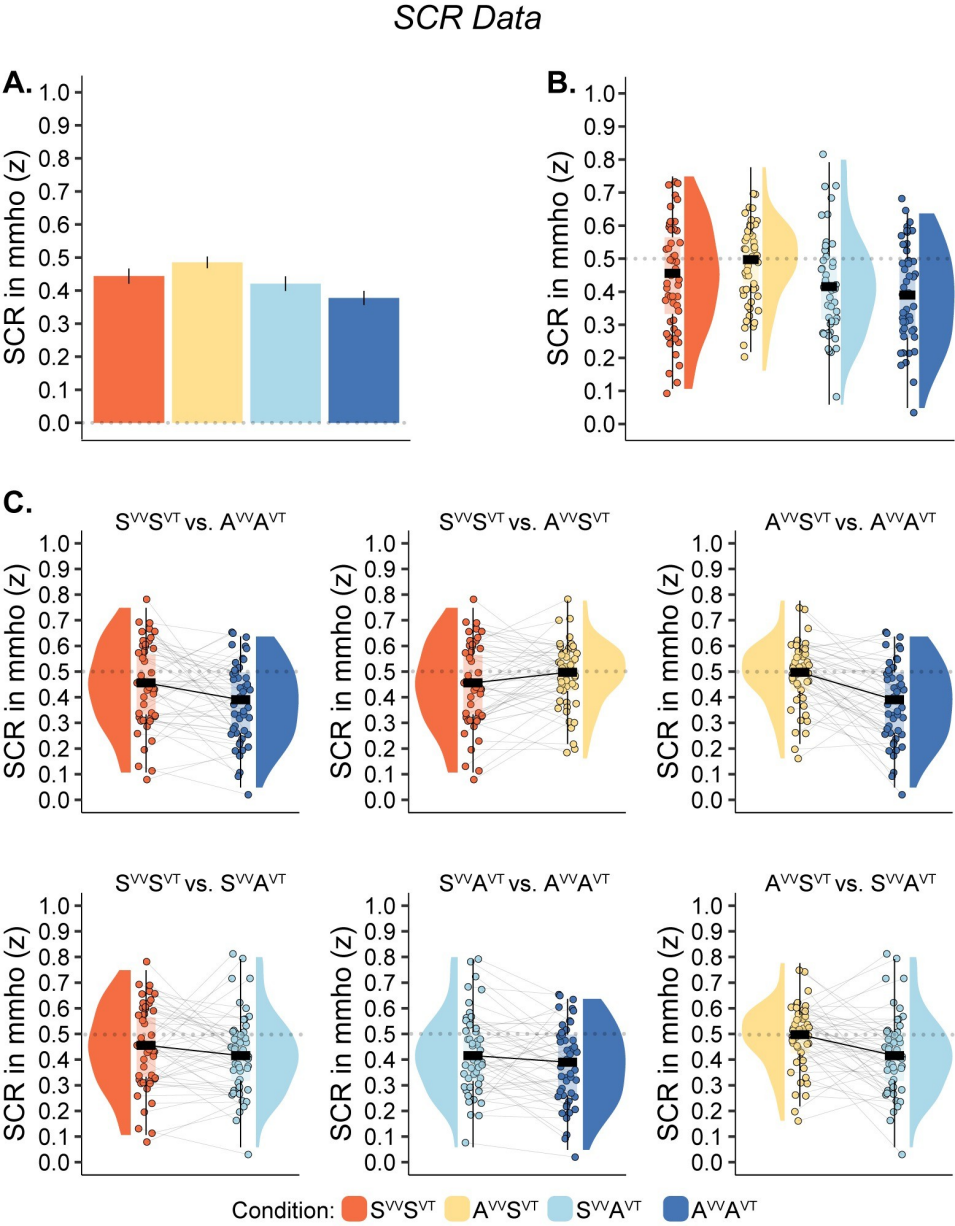
Plots show normalized SCR magnitude data (N = 50). (**A**). Barplot shows means and standard errors for illustrative purposes. (**B**). Raincloud plots show individual data points and medians. (**C**). Raincloud plots for SCR magnitude data. Individual data points, medians, paired lines, and distributions are displayed.

Planned comparisons between the three conditions where some degree of illusion was expected (S^VV^S^VT^, A^VV^S^VT^ and S^VV^A^VT^) and the fully asynchronous condition when the illusion was expected to be eliminated (A^VV^A^VT^) showed significant differences in all three cases: S^VV^S^VT^ > A^VV^A^VT^, *t* = 2.583, *p* = 0.006, BF_10_ = 6.028; A^VV^S^VT^ > A^VV^A^VT^, *t* = 4.561, *p* < 0.001, BF_10_ > 100; S^VV^A^VT^ > A^VV^A^VT^, *t* = 2.052, *p* = 0.023, BF_10_ = 2.053. No higher SCR was found for S^VV^S^VT^ than for A^VV^S^VT^ (*t* = -1.983, *p* = 0.974, BF_10_ = 0.055) or between S^VV^S^VT^ and S^VV^A^VT^ (*t* = 0.951, *p* = 0.173, BF_10_ = 0.387). All results are summarized in Table 3 and illustrated in Fig 4.

**Table 3:** Skin conductance response magnitude results (N = 50). We tested the one–sided hypotheses that S^VV^S^VT^ > A^VV^S^VT^, S^VV^S^VT^ > S^VV^A^VT^, S^VV^S^VT^ > A^VV^A^VT^, A^VV^S^VT^ > A^VV^A^VT^ and S^VV^A^VT^ > A^VV^A^VT^ and the two–sided hypothesis that S^VV^A^VT T^≠ A^VV^S^VT^. “t” shows the t–statistics derived from t tests, “p” the p value, “BF_10_” the Bayesian factor for the alternative hypothesis, and “d_z_” the Cohen’s coefficient for matched pairs as effect size.

*Comparison*	*t*	*p*	*Tails*	*BF*_*10*_	*Effect size (d*_*z*_ *)*
S^VV^S^VT^ > A^VV^S^VT^	-1.983	0.974	1	0.055	-0.28
S^VV^S^VT^ > S^VV^A^VT^	0.951	0.173	1	0.387	0.134
S^VV^S^VT^ > A^VV^A^VT^	2.583	0.006**	1	6.028	0.621
A^VV^S^VT^ > A^VV^A^VT^	4.561	< 0.001***	1	> 100	0.645
S^VV^A^VT^ > A^VV^A^VT^	2.052	0.023*	1	2.053	0.329
S^VV^A^VT^ ≠ A^VV^S^VT^	-2.72	0.009**	2	4.113	-0.385

Note: *** < 0.001,

**< 0.01,

*< 0.05

In ‘Supporting Information—Section III’ in S1 File, we report the results from three complementary analyses where we reanalyzed the SCR magnitude data without normalization and/or when excluding the two participants with very weak SCR responses (the “null responders”). The results were very similar to the main analysis described above, with all statistically significant comparisons between the conditions still being statistically significant in the three extra analyses; the only noteworthy difference was that the order effect was no longer significant.

### Bayesian causal inference model

**Fig 5:**
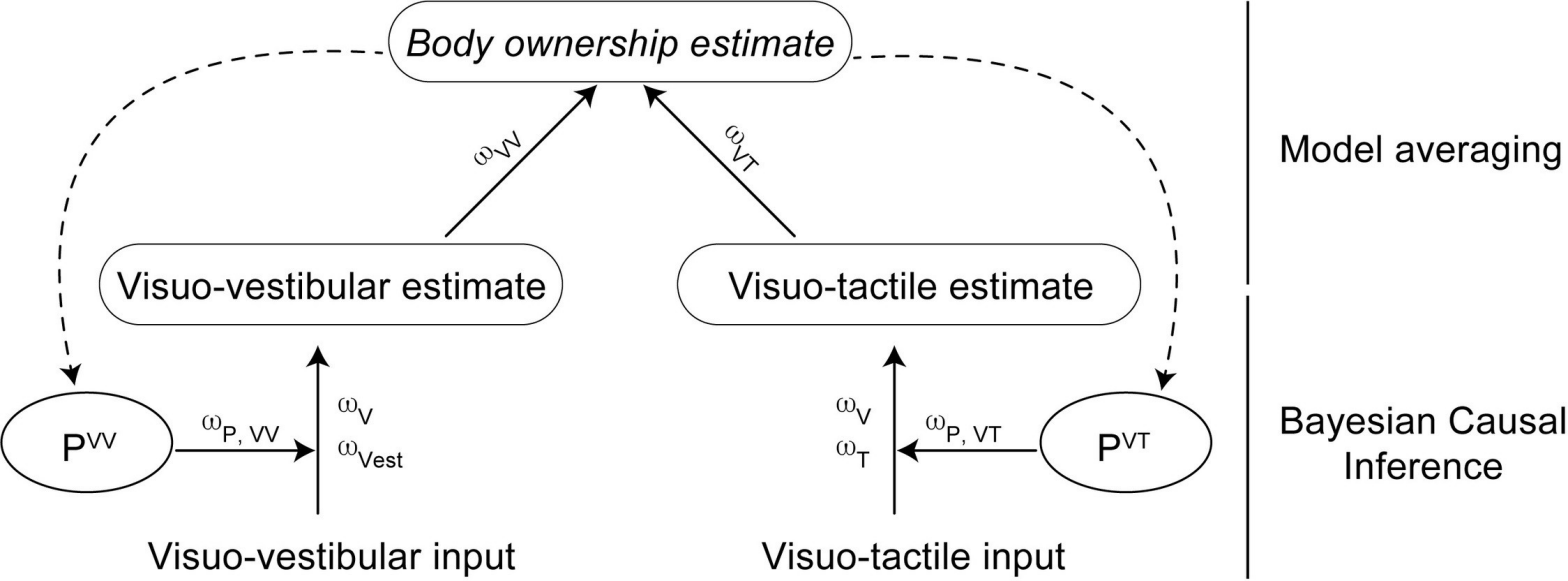
Theoretical model for the integration of visual, tactile, and vestibular signals for body ownership based on Bayesian probabilistic principles. For each stimulation, the pair of signals (visual and vestibular; visual and tactile) are integrated according to their causal structure inferred based on their relative reliability (ω_V_, ω_Vest_, ω_T_) and the prior probability for them to emerge from a common cause (P_VV_, P_VT_); for each bimodal pair, the influence of the a priori probability for a common cause (bisensory priors) on the causal inference process is weighted by the relative reliability of this a prior signal (ω_P,VV_, ω_P,VT_). The resulting visuo–vestibular and visuo–tactile estimates are then combined, again according to their relative reliability (ω_VV_, ω_VT_). In turn, the emerging body ownership percept influences the bisensory priors. Such a model matches our results when assuming ω_VV_ ≪ ω_VT_.

We developed a Bayesian causal inference model to describe how the visuo-tactile and visuo-vestibular correlations both contribute to the body ownership illusion and self-motion but to different degrees, as the questionnaire data showed. This was an extension of the model described in detail in [17]. The central idea in the current extended model is that the automatic perceptual decision to integrate rather than segregate the visual, tactile and vestibular information into a coherent perceptual experience of one’s own body “swinging” in space can be described as a dynamic two-level hierarchical causal inference process with three key components. Visual and tactile information and visual and vestibular information are first combined according to the relative reliability of the sensory signals and the prior probability of a common cause in two separate causal inference processes. The resulting estimates from these two processes are then combined at the second level and weighted according to their relative reliability, giving rise to the overall experience of ownership of the mannequin’s body. In turn, this ownership percept emerging at the second level affects each of the two bisensory causal inference processes at the first level by influencing the prior probabilities for a common cause. The model is presented in Fig 5.

**Fig 6:**
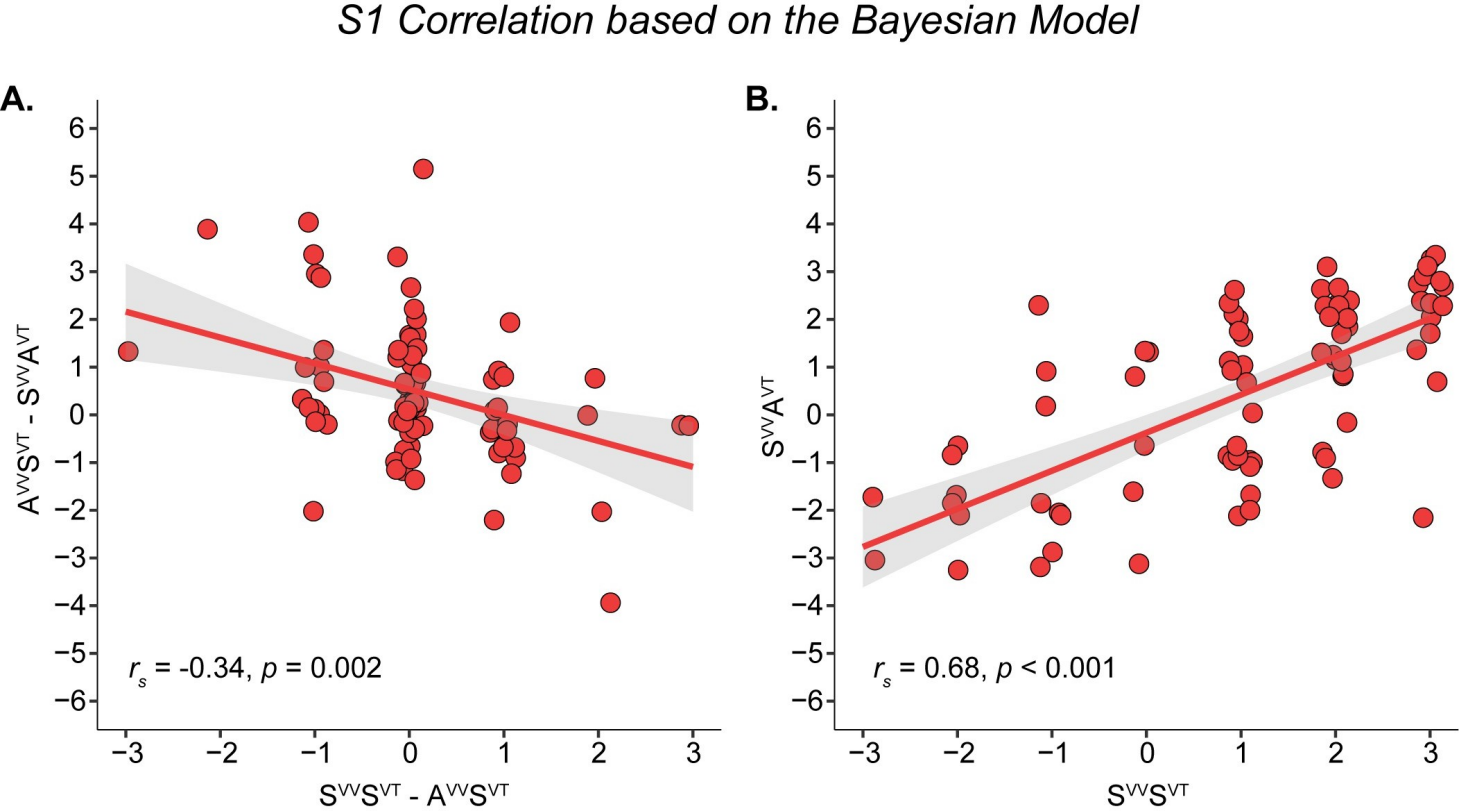
Correlational analyses in support of the proposed Bayesian model. (**A**) Correlation analysis of the difference in questionnaire statement S1 for A^VV^S^VT^ minus S^VV^A^VT^ and the corresponding S1 difference S^VV^S^VT^ minus A^VV^S^VT^ (N = 80). A linear regression plot is shown for illustrative purposes. This plot shows that, as expected and in agreement with our theoretical model, the greater the visuo–vestibular asynchrony hinders the illusion compared to the fully synchronous condition (x–axis: S^VV^S^VT^–A^VV^S^VT^), the weaker the relative dominance of the visuo–tactile information over the visuo–vestibular information (y–axis: A^VV^S^VT^–S^VV^A^VT^). (**B**) Positive correlation between S^VV^A^VT^ and S^VV^S^VT^, which further supports the proposed Bayesian model by confirming that the significant correlation shown in A is not driven by condition A^VV^S^VT^ that is used to calculate both difference scores. The Spearman correlation was r_s_ = 0.68, ^95%^CI: 0.52–0.8, p < 0.001.

A prediction from this model is that the *stronger* the feeling of ownership (S1) in the fully synchronous condition (S^VV^S^VT^) compared to the condition where only the visuo-vestibular condition is asynchronous (A^VV^S^VT^), the *weaker* the ownership illusion should be in the condition with asynchronous visuo-vestibular and synchronous visuo-tactile stimulation (A^VV^S^VT^) compared to the condition with synchronous visuo-vestibular and asynchronous visuo-tactile stimulation (S^VV^A^VT^). To examine whether our questionnaire data were in line with this prediction, we conducted an explorative post hoc correlation analysis. As expected from the model, we found a negative correlation between the S1 difference score for A^VV^S^VT^ minus S^VV^A^VT^ and the S1 difference score for S^VV^S^VT^ minus A^VV^S^VT^ (*r*_*s*_ = -0.34, *p* = 0.002; Fig 6A). In other words, the *stronger* the effect of visuo-vestibular congruence on body ownership (S^VV^S^VT^ minus A^VV^S^VT^), the weaker the relative dominance of visuo-tactile congruence over visuo-vestibular congruence (A^VV^S^VT^ minus S^VV^A^VT^). This statistical correlation is in line with a dynamic relationship in the relative weightings of the two bisensory causal inference processes on the multisensory body ownership experience arising at the top level in the model. To rule out the possibility that this finding was driven by variability in the A^VV^S^VT^ rather than in the difference scores, we also examined whether the S1 ratings in S^VV^S^VT^ and S^VV^A^VT^ were positively correlated, as we would expect them to be, and indeed this was the case (*r*_*s*_ = 0.68, *p* < 0.001; Fig 6B).

## Discussion

The aim of the present study was to investigate visuo-vestibular and visuo-tactile contributions to body ownership in a single experimental design with a full-body illusion. There were three main findings. First, and as expected, we observed that synchronous visuo-tactile and synchronous visuo-vestibular information resulted in higher ownership ratings than the case when both visuo-tactile and visuo-vestibular information were asynchronous. In line with this, the results of the SCR analysis revealed that all three conditions that included at least one bimodal synchronous stimulation elicited stronger threat-evoked physiological responses than the fully asynchronous condition. Second, we found that the visuo-tactile correlations dominated over the visuo-vestibular correlations so that synchronous visuo-tactile stimulation combined with asynchronous visuo-vestibular stimulation elicited a subjective ownership illusion that was similarly strong as the condition when both pairs of modalities were stimulated synchronously. Finally, both visuo-tactile and visuo-vestibular temporal congruence boosted the sense of self-motion, supporting the previous claim [30] that body ownership boosts self-motion perception by making the body-related visual cue for self-motion more potent. Collectively, these results are interesting because they imply that the brain combines evidence from different combinations of sensory correlations to generate coherent (illusory) own-body experiences and self-motion sensations. When one combination of bimodal stimuli is incongruent, illusory body ownership can still be elicited if there is strong evidence from another set of congruent bimodal stimuli speaking in favor of body ownership. As we will argue further below, these results can be explained in a flexible probabilistic model of multisensory perception of one’s own body.

Previous studies on full-body ownership focused on visuo-tactile multisensory integration [10, 36, 40, 55]. During these studies, the vestibular information was kept constant, and neither GVS nor head movements were manipulated. In a very recent study, we conducted three experiments in which participants were only exposed to visuo-vestibular synchronous or asynchronous stimulation without visuo-tactile stimulation. Here, we were able to show that visuo-vestibular stimulation is sufficient to induce a full-body ownership illusion [16]. This previous study provided evidence for a significant effect of visuo-vestibular temporal congruence on the strength of the full-body illusion. The results from the current study are consistent with those of [16], who showed an effect of visuo-vestibular congruence in both questionnaires and SCR. However, the current factorial design also revealed an asymmetry between the visuo-vestibular and visuo-tactile temporal rules. More specifically, the present results show that ownership ratings were significantly higher in the visuo-tactile synchronous condition that included asynchronous visuo-vestibular stimulation (A^VV^S^VT^) than in the visuo-vestibular synchronous condition that included asynchronous visuo-tactile stimulation (S^VV^A^VT^). Furthermore, there was ‘anecdotal’ evidence in favor of the null hypothesis in ownership ratings when comparing the condition with visuo-tactile synchrony and visuo-vestibular asynchrony (A^VV^S^VT^) to the condition with both visuo-tactile and visuo-vestibular synchronies (S^VV^S^VT^) (*p* = 0.158, BF_01_ = 2.955, BF_10_ = 0.338). In contrast, when comparing the two conditions with synchronous visuo-vestibular stimulation where only visuo-tactile temporal congruence was manipulated (S^VV^A^VT^ to S^VV^S^VT^), we found that asynchronous visuo-tactile stimulation resulted in a significant reduction in the ownership ratings. However, no corresponding effect of visuo-vestibular asynchrony leading to a statistically significant reduction in ownership ratings was observed when comparing the two conditions with synchronous visuo-tactile stimulation (A^VV^S^VT^ to S^VV^S^VT^). These findings suggest that although both visuo-tactile and visuo-vestibular correlations contribute to the full-body illusion when the body is viewed from the first-person perspective, visuo-tactile temporal correlations are more dominant, at least in the current version of the paradigm.

Analysis of the threat-evoked SCR data revealed that all synchronous stimulation conditions (S^VV^S^VT^, A^VV^S^VT^, S^VV^A^VT^) increased the SCR magnitude compared to the asynchronous A^VV^A^VT^ condition (in the linear mixed model), in line with the illusion being influenced by the temporal congruence rule in both pairs of sensory modalities. Furthermore, and consistent with the questionnaire findings, the pairwise comparisons of SCR magnitude revealed that there was a significant difference between A^VV^S^VT^ and S^VV^A^VT^, showing higher SCR magnitude in A^VV^S^VT^, and no significant difference between S^VV^S^VT^ and A^VV^S^VT^; two findings that both are in line with a relatively greater effect of visuo-tactile correlations. Notably, in the A^VV^S^VT^ condition, synchronous visuo-tactile information is associated with a similar degree of threat-evoked SCR magnitude as in the fully synchronous S^VV^S^VT^ condition, even if asynchronous visuo-vestibular information is provided in the former case. However, in contrast to the questionnaire findings, we did not find a significant difference between S^VV^S^VT^ and S^VV^A^VT^ in SCR magnitude. Therefore, the SCR results do not support the hypothesis that asynchronous visuo-tactile stimulation is more effective in breaking the illusion, which was observed in the questionnaire data. However, it is unclear how sensitive the threat-evoked SCR procedure is to detect differences in the degree of body ownership perception, and most previous studies that have used this physiological measure have used it to compare conditions when a vivid illusion is compared to a control condition where the illusion is eliminated or strongly suppressed [10]. We speculate that it is possible that even if the full-body illusion is slightly reduced on one condition compared to another but still felt to a certain degree (affirmative illusion ratings), it could trigger an automatic emotional defense against physical threats. However, further methodological work is needed to investigate the precise relationship between changes in subjective ownership illusion and graded responses in threat-evoked SCR. For example, in the current data, we found no significant correlation between questionnaire rating S1 and the magnitude of the threat-evoked SCR response with regard to the different measures for S^VV^S^VT^ and A^VV^A^VT^. Therefore, we focus more on the questionnaire data in the current study and consider the SCR findings supplementary, mainly supporting that a bodily illusion was elicited in the S^VV^S^VT^, A^VV^S^VT^, and S^VV^A^VT^ conditions compared to the fully incongruent condition (A^VV^A^VT^).

A further interesting result is that visuo-tactile synchrony also induced a stronger sensation of self-motion (S7), in support of our hypothesis. This finding is in line with the results of a previous study in which we showed that visuo-tactile synchrony influences self-motion perception and orientation perception [30]. In this previous study, a visuo-tactile full body ownership illusion was combined with a reorientation illusion, where a fully furnished virtual room was presented to the participants through an HMD. This room was then rotated to induce a sensation of self-motion and reorientation (‘a feeling of being upside-down’) in participants. The results showed that feelings of self-motion and reorientation were stronger when illusory body ownership was induced by concurrent synchronous visuo-tactile stimulation than by an asynchronous control. Importantly, the present results not only confirm that visuo-tactile synchrony boosts visually induced self-motion perception but also add two significant observations. First, the augmentation of self-motion perception driven by visuo-tactile synchrony adds to the effect of visuo-vestibular synchrony so that the greatest self-motion occurs when both types of bimodal correlations are present (Fig 3). The augmented effects of visuo-tactile and visuo-vestibular synchrony seem to be comparable since the self-motion ratings were similar in A^VV^S^VT^ and S^VV^A^VT^. Second, synchronous visuo-tactile stimulation also led to stronger visuo-vestibular perceptual binding (S2)–such that the body motion seen was the body motion felt–even when asynchronous visuo-vestibular stimulation was delivered. This again illustrates how the effect of congruent visuo-tactile stimulation can ‘overwrite’ temporal incongruent vestibular information in the full-body illusion. Presumably, these effects on self-motion and visuo-vestibular binding come from the increased body ownership perception elicited by synchronous visuo-tactile stimulation. The enhanced ownership of the body in view probably made the visual impression of the environment rotating around the body more potent as a self-motion cue.

To account for both main findings discussed above, the stronger effect of visuo-tactile asynchrony than visuo-vestibular asynchrony in suppressing the ownership illusion and the enhancing effect of visuo-tactile synchrony on self-motion perception, we propose a model based on the Bayesian causal inference theory of multisensory integration [57]. This theory provides a solution to the problem of deciding which sensory signals should be combined and which should be segregated in the process of creating coherent multisensory percepts of objects and events [58–60] and, more recently, the body [17, 18, 61, 62]. In causal inference models, the most likely causal structure of multiple sensory events is estimated based on spatiotemporal correspondence, sensory uncertainty, and prior experiences. Moreover, the Bayesian framework assumes that sensory inputs and prior beliefs regarding the causal structure of the multisensory event contribute to the resulting perception with respect to their relative reliability. This Bayesian optimal behavior has been observed when integrating vision and touch for the experience of one’s own body [17, 18] and when integrating vision and vestibular information for self-motion perception [63, 64].

Thus, we propose that in our experiment, visual and vestibular signals and visual and tactile signals are integrated according to the Bayesian causal inference principle, leading to a visuo-vestibular estimate and a visuo-tactile estimate, respectively. Then, the optimal body ownership estimate will be a weighted average of the two causal models, which is called model averaging [18]. This implies that the optimal estimates will in most cases include influences of both causal models, again with respect to their relative reliability (Fig 5). The tactile impressions of the probe touching the abdomen are more salient, vivid, and perceptually distinct than the GVS-induced movement sensations that are somewhat less vivid and vaguer in terms of precise onset and offset (although synchrony and asynchrony can be clearly perceived). Consequently, the visuo-tactile estimate, relatively more reliable, contributes to the body ownership percept to a larger extent than the visuo-vestibular estimate. Finally, the resulting body ownership estimate may in turn shape the a priori probability of integrating visual and vestibular signals as well as vision and touch. This model would explain the influence of visuo-tactile congruence on visuo-vestibular binding (S2 of the subjective ratings) and self-motion perception (S7). Our model also predicts an influence of visuo-vestibular congruence on visuo-tactile binding; however, the likely relatively higher reliability of the visuo-tactile inputs (see below) would explain the absence of a significant effect in our observations.

Whether the different impacts of the visuo-tactile and visuo-vestibular correlations in the current study relate to fundamental differences in how these two different pairs of sensory modalities are integrated or to the particular stimulus parameters used in the present study is unclear and deserves further investigation in future studies. Is visuo-tactile input more important for the sense of body ownership than visuo-vestibular evidence? From the perspective of Bayesian causal inference, the question is ill-posed because it is not the sensory modalities themselves that are critical or a certain modality that “dominates” but the information they carry that is relevant for the perceptual decision process, and this can vary depending on context, prior knowledge and a variety of factors related to the particular stimulus parameters and their spatiotemporal relationships in a quite flexible way. In the current experiment, visuo-vestibular stimulation was achieved through artificial electrical stimulation of the vestibular nerve through the GVS, which might have compromised the reliability of the vestibular input compared to more ecological stimuli, we speculate. This might explain why asynchronous visuo-vestibular information appears to be unable to break an ownership illusion induced through visuo-tactile synchronous sensory information. The tactile stimulation applied by stroking the participants’ bodies with a physical object might have generated a more precise, less noisy and therefore more informative cue. If the tactile cues are more precise and reliable than the vestibular cues and the visual stimuli are always constant, then the visuo-tactile congruence effect on the full-body illusion would be stronger than the visuo-vestibular congruence effect, we theorize. Similarly, the visuo-tactile stimuli were applied at a higher frequency (approximately 0.5 Hz) compared to the visuo-vestibular stimuli (approximately 0.15 Hz), meaning that there were more bimodal events in the visuo-tactile correlations; thus, the information from the visuo-tactile correlations would provide more reliable information. Indeed, we know from a rubber hand illusion experiment that increasing the information content in visuo-tactile correlations boosts hand-ownership illusion during simultaneous visuo-proprioceptive conflict [65]. In our Bayesian causal model, less reliable visuo-vestibular information due to the artificial GVS stimulation or more reliable visuo-tactile information due to more visuo-tactile events in the correlations would be mathematically equivalent, leading to more reliable visuo-tactile estimates that therefore influence the overall body ownership estimate more than the less reliable visuo-vestibular estimates (Fig 5). Future studies should further test this model by either making the vestibular information more reliable or decreasing the reliability of the visuo-tactile information. This could be done by, for example, blurring the visual stimuli, replacing the natural brushstrokes with weak vibratory stimulus, replacing the GVS stimulation with actual whole-body rotations using a motion platform, or presenting the visuo-tactile stimuli at a slower frequency than the visuo-vestibular ones. An alternative possibility is that when there is a conflict between visuo-tactile and visuo-vestibular combinations of bimodal sensory information (such as during A^VV^S^VT^ and S^VV^A^VT^), a simple strategy of cue integration might be to downweigh input from visuo-vestibular stimulation [66] and rely more on visuo-tactile integration. Such a “fixed-criteria” strategy could in principle also explain the current results and be more in line with the idea of visuo-tactile “dominance”, but the results from our post hoc analysis are more in line with a probabilistic framework. Accordingly, we found that the *greater* difference in ownership illusion (S1) between the fully synchronous condition (S^VV^S^VT^) and the condition with asynchronous visuo-vestibular stimuli and synchronous visuo-tactile stimuli (A^VV^S^VT^) (that is S^VV^S^VT^ minus A^VV^S^VT^), the *smaller* the difference in illusion strength (S1) between the condition with asynchronous visuo-vestibular stimuli and synchronous visuo-tactile stimuli (A^VV^S^VT^) and the condition with synchronous visuo-vestibular and asynchronous visuo-tactile condition (S^VV^A^VT^) (that is A^VV^S^VT^ minus S^VV^A^VT^). This statistical correlation (see Fig 6A) suggests that the more reliable the visuo-vestibular estimates are, meaning that the visuo-vestibular correlations have a greater impact on the ownership illusion, the more similar the two bimodal estimates are in terms of reliability, and, therefore, the more similar the two sets of bimodal correlations are in driving the illusion, which is what the Bayesian causal inference model predicts (Fig 5). A “fixed criteria” strategy of always relying more on the visuo-tactile correlations and downweighing the visuo-vestibular evidence should not produce this correlation (Fig 6). Finally, we should point out that the current causal inference model was designed with the current experiments and sensory modalities in mind, but the model could be expanded to include other combinations of sensory modalities, such as visuo-proprioceptive information during limb movement [9] or visuo-interoceptive cues from seeing and feeling breathing movements of the chest [13]. Such expanded or modified versions of the current model may help to provide a new understanding of some interesting previous observations in the previous literature on body ownership illusions where certain sets of multisensory cues seem to “dominate” other cues. For example, congruent visuo-proprioceptive signals from movement and posture can elicit a vivid full-body illusion even when asynchronous visuo-tactile stimulation is delivered under certain conditions [9], and adding congruent signals in an extra sensory channel does not always lead to a significantly stronger illusion as in the observation that CT-optimal affective touch stimulation (a skin-based interoceptive submodality) did not boost the full-body ownership illusion over and above congruent “nonaffective” tactile stimulation [11]. Thus, we theorize that the way different combinations of sensory modalities are combined and drive body ownership can vary in different paradigms depending on the relative reliability and information content of the various bisensory stimuli and their correlations. In the current experiments and model, proprioceptive input was kept constant in all conditions (no movements, immobile relaxed), so the influence from this modality was expected to be less pronounced and therefore not included in the model, but information from this modality also makes an important contribution to body ownership [8, 18, 61, 67], and there are important interactions between proprioceptive feedback from the neck region and vestibular signals during head movements [68, 69] that could be interesting to investigate in the context of body ownership and bodily illusion in future studies.

In conclusion, the present study provides evidence that the temporal rule of multisensory integration applies to both visuo-vestibular and visuo-tactile combinations of sensory stimulation in the full-body ownership illusion when the artificial body is viewed from the first-person perspective. However, in the current study, visuo-tactile synchrony had a stronger influence on the ownership illusion than visuo-vestibular synchrony when both were applied simultaneously, which we interpret as being due to differences in the relative importance of the two types of temporal correlations in causal inference and multisensory integration. Future neuroimaging studies should investigate how the brain creates a unified experience of one’s whole body by integrating congruent visuo-tactile [36, 55] and congruent visuo-vestibular information and identify the associated cortical convergence areas where the three-way multisensory interactions of vision, touch, and vestibular sensations are implemented at the neuronal population level.

## Supporting information

S1 File Pilot experiment 2s, extra figures and tables, and extra SCR analyses. (DOCX)

S1 Data Source data. (XLSX)
