# Supplementary material for: Combination of visuo-tactile and visuo-vestibular correlations in illusory body ownership and self-motion sensations
Source: PLoS One. 2022 Nov 15;17(11):e0277080. doi: 10.1371/journal.pone.0277080 (PMC9665377; doi:10.1371/journal.pone.0277080)
Supplement: S1 File — (DOCX) [file pone.0277080.s001.docx]

Section I – Pilot experiment

**Pilot SCR Experiment**

The pilot experiment 2s was conducted before the main study described in the article and served to validate the experimental paradigm and the effectiveness of the new visual threat stimuli used in skin conductance response (SCR) recordings.

**Methods**

*Participants*

We recruited a total of 26 participants. One participant was excluded from the analysis because of low task compliance; therefore, we analyzed data from 25 (17 male, mean age=28.08, SD=6.27). All participants, except two, were identified as right-handed as indicated by the Edinburgh Handedness Questionnaire [1]. All participants had normal or corrected-to-normal vision and gave written informed consent. The experimental procedure was approved by the Swedish Ethics Review Authority.

*Stimuli and Apparatus*

The video stimulus material was identical to the main SCR experiment, with the exception that the video contained no visual self-motion stimuli and the participants did not receive galvanic vestibular stimulation (GVS).

*Procedure*

The procedure for the pilot experiment was comparable to the procedure described for the main SCR experiment described in the article. Participants were lying on a bed with their head tilted forward approximately 45 degrees. The head position was stabilized with a pillow that was taped to the bed. Video material was presented using a head mounted display (HMD) (Oculus Rift 2, <http://www.coulusvr.com/>). The experiment consisted of two different conditions: visuo-tactile synchrony and visuo-tactile asynchrony. Participants watched 16 short videos (length between 61 and 67 sec) where they were either exposed to synchronous (S^VT^) or asynchronous visuo-tactile stimulation (A^VT^). The stimulation condition was alternated, and half of the participants started with synchronous stimulation, whereas the other half started with asynchronous stimulation. Each condition lasted between 59 and 67 sec, and participants had to complete five questionnaire statements afterward to measure subjective experience of illusory ownership (S1), illusory touch on the mannequin (S2) and various control statements (S3-S4) (S1 Table). Importantly, 16 different threat stimuli involving different kinds of sharp and blunt tools making “slicing”, “stabbing” and “hammering” movements toward the mannequin were used (see Fig 1 of the main article). Instead of a single knife threat as in the study by Petkova & Ehrsson [2] , this variety of techniques was used, and SCR was registered, serving as an objective measurement for the body ownership illusion. Hence, one knife threat per video was presented, and knife threats were applied randomly between seconds 39 and 64. The duration of each threat was approximately 1 sec.

| Statement | During the experiment: | Type |
| --- | --- | --- |
| S1 | …it felt as if I was looking at my body. | Body ownership illusion |
| S2 | … it seems as though the touch I felt was caused by the white ball. | Visuo-vestibular binding |
| S3 | … it felt as if I had two bodies. | Visuo-tactile binding |
| S4 | … I felt as if my body was turning ‘plastic’ | Illusion control |
| S5 | … I felt dizzy. | Illusion control |

***S1 Table. Illusory Ownership Questionnaire****.*

*Analysis*

When data were not normally distributed (Shapiro‒Wilk p value <.05), we ran Wilcoxon signed-rank tests and reported the matched-pairs biserial correlation as effect size (“*r_C_*”; [3,4]). In contrast, when data were normally distributed (Shapiro‒Wilk p value >.05), we ran t tests and reported *Cohen’s d_z_* as effect sizes. In addition, we report the achieved power for the SCR magnitude analysis using the package “pwr” [5]. We report Bayes factors (BF_10_) in favor of the alternative hypothesis for each comparison.

Questionnaire data were analyzed using Wilcoxon signed-rank tests. Questionnaire statements S1 and S2 were tested one-sided, as we had strong hypotheses about the direction of the effect [2]. The control statements were tested two-sided, instead. The SCR magnitude data were normalized around the highest response of each subject, as in the main experiment. SCR data were normally distributed, and since we had a strong hypothesis of higher SCR under the synchronous condition than under the asynchronous condition, we analyzed them with a one-tailed t test.

**Results**

The questionnaire results are summarized in S2 Table and S1 Fig. Importantly, these show a significantly greater score in the synchronous visuo-tactile stimulation condition compared to the asynchronous condition for the body ownership statement S1 and the referral of touch statement S2 (S1: *V* = 82, *p* = 0.005, BF_10_ = 8.972, *r_C_* = 0.802; S2: *V* = 101.5, *p* = 0.009, BF_10_ = 5.685, *r_C_* = 0.692). None of the control statements were significant when we compared the synchronous and asynchronous conditions (*p* > 0.19).

| ***Statement*** | ***Comparison*** | ***V*** | ***p*** | ***tails*** | ***BF_10_*** | ***r_C_*** |
| --- | --- | --- | --- | --- | --- | --- |
| S1 | S^VT^ > A^VT^ | 82 | 0.005** | 1 | 8.972 | 0.802 |
| S2 | S^VT^ > A^VT^ | 101.5 | 0.009** | 1 | 5.685 | 0.692 |
| S3 | S^VT^ ≠ A^VT^ | 27.5 | 0.218 | 2 | 0.110 | -0.396 |
| S4 | S^VT^ ≠ A^VT^ | 44 | 0.347 | 2 | 0.545 | 0.333 |
| S5 | S^VT^ ≠ A^VT^ | 55.5 | 0.202 | 2 | 0.491 | 0.423 |

***S2 Table.*** ***Results of the questionnaire data Experiment 2s*** *(N = 25).*

*Note: *** < 0.001, **< 0.01, *< 0.05*


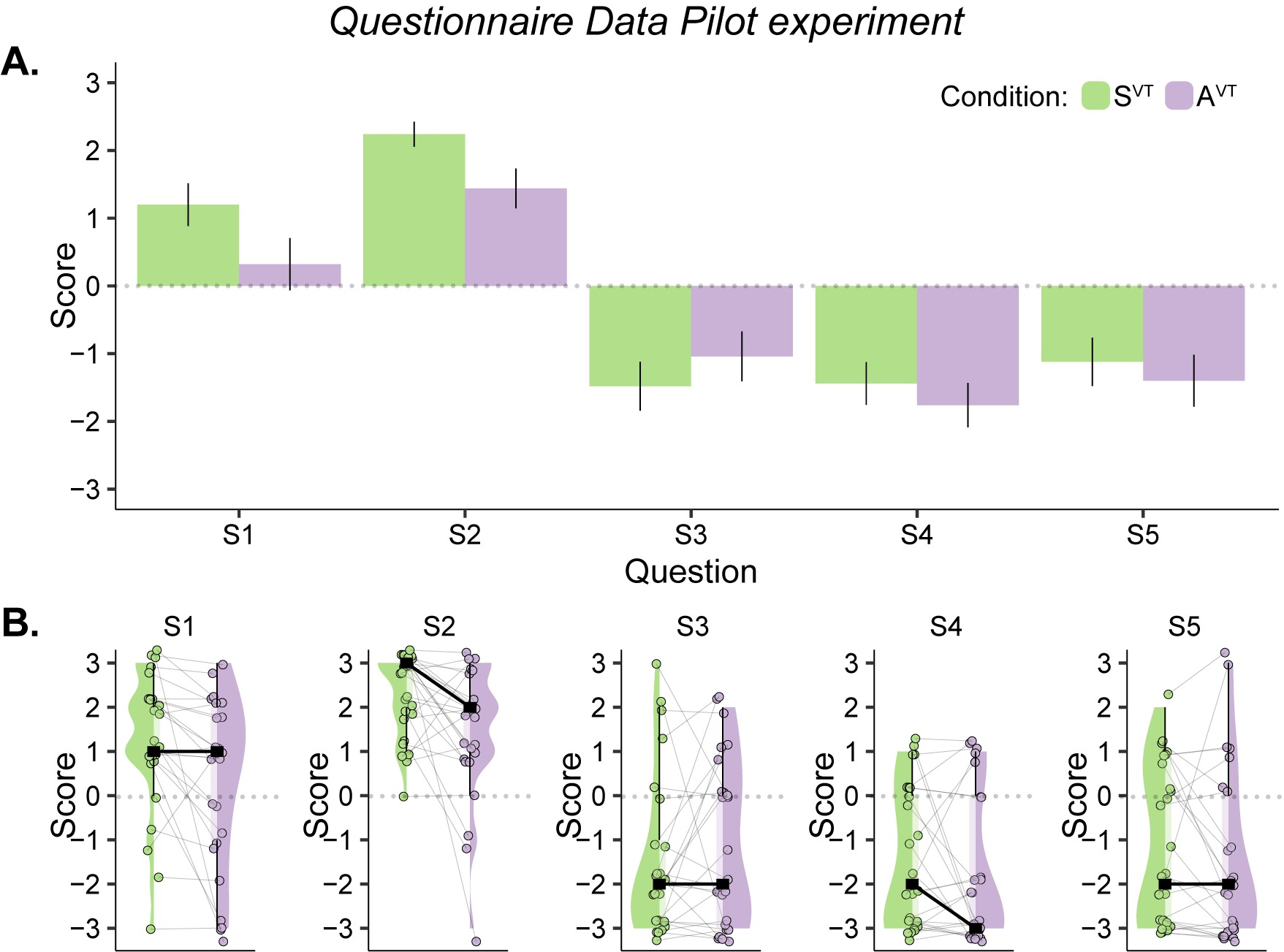


***S1 Fig.*** ***Questionnaire results of the pilot experiment (N=25).*** ***A.*** *Barplot showing the means and standard error for illustrative purposes.* ***B.*** *Raincloud plots show individual data points, medians, paired lines and distributions for each statement in the questionnaire.*

The SCR magnitude data were normalized around the highest response of each subject. Analysis of the SCR magnitude data revealed a significant effect from the condition: SCR was higher during synchronous than during asynchronous visuo-tactile stimulation (*t* = 1.77, *p* = 0.045, BF_10_ = 1.552, *d_z_* = 0.354) (S2 Fig). The effect size was 0.354, indicating a ‘medium’ effect [6]. The power analysis revealed an achieved power of 0.341. A greater power of 0.8 would have required 50 participants.


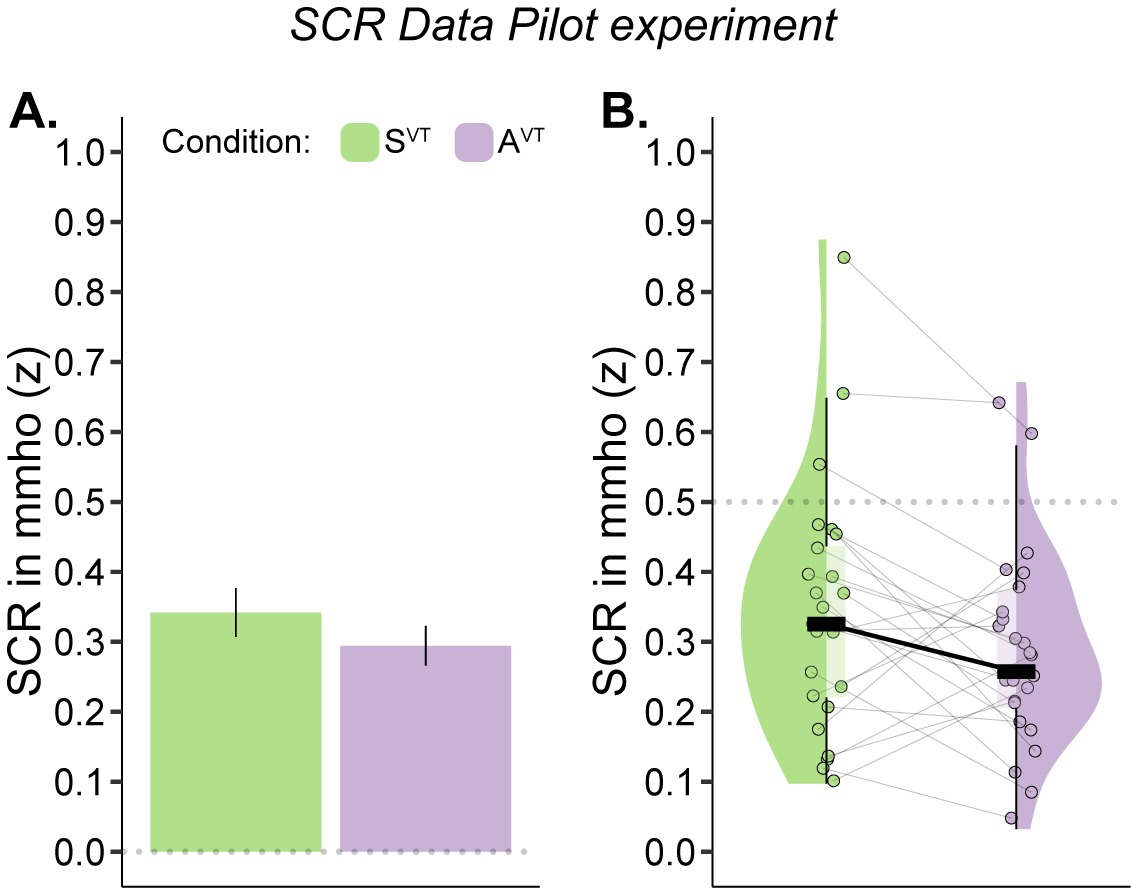


***S2 Fig.*** ***Skin conductance response (SCR).*** *SCR to knife threats toward the mannequin’s body in the SCR pilot experiment (N=25).*

**Summary**

The pilot experiment served to verify the new threat stimulus we used in the SCR experiment in the main study. As expected from previous studies, we found a significant effect of synchronous visuo-tactile stimulation on ownership ratings and threat-induced SCR. The results of this pilot study hence show that our basic full-body illusion paradigm with visuo-tactile stimulation worked as expected and that the new visual threat stimuli were effective for SCR. To achieve greater statistical power, we doubled the sample size in the main experiment. As reported in the main text, we also used to present questionnaire data from statement S1 to compare the strength of illusory body ownership in bimodal visuo-tactile and trimodal visuo-vestibular-tactile stimulation conditions.

Section II – Supplementary Images and Tables


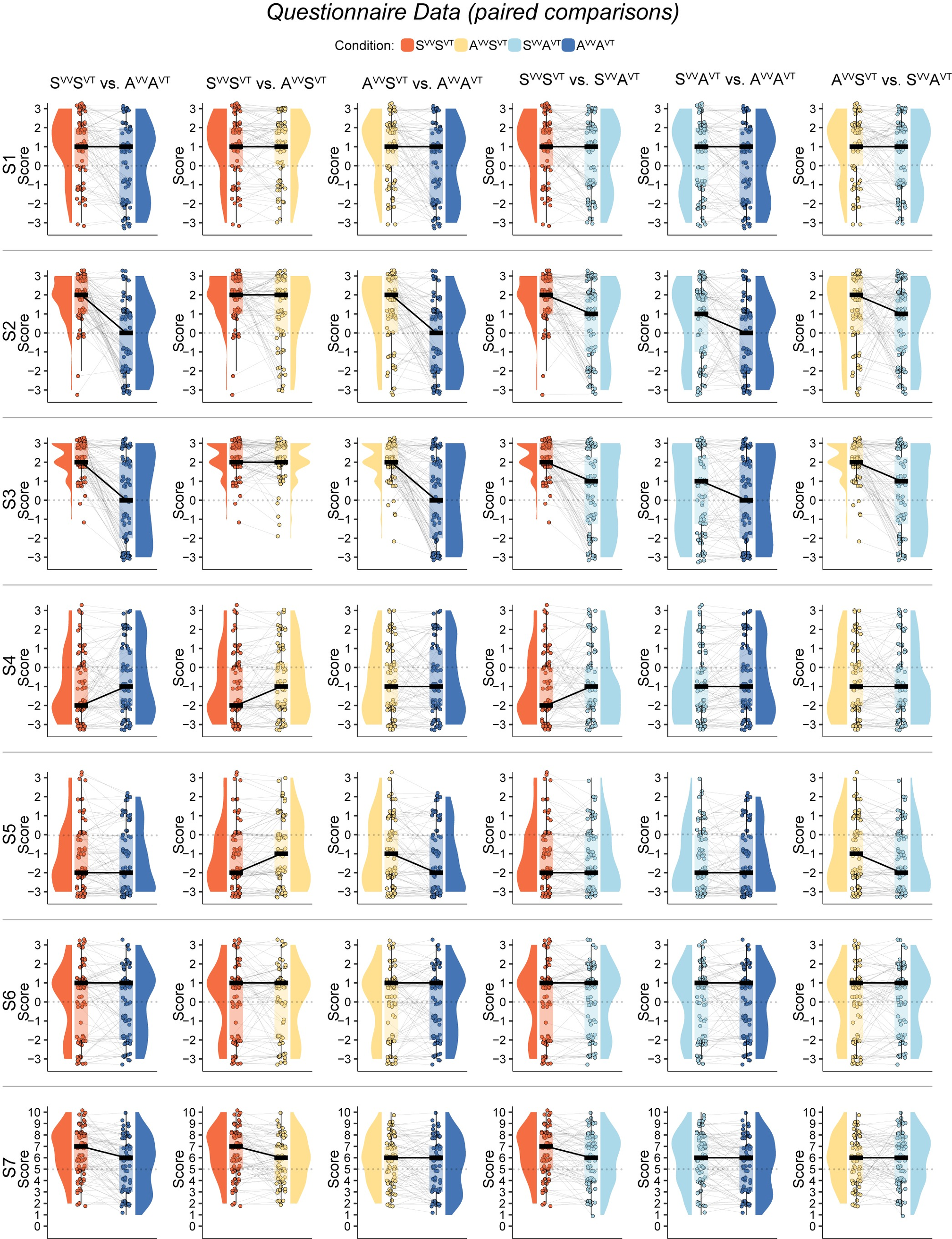


***S3 Fig.*** *Raincloud plots for each question and each comparison (N=80). Individual data points, medians, paired lines and distributions are displayed.*

**Descriptive statistics for the questionnaire data**

| ***Question*** | ***Condition*** | ***Mean (±SD)*** | ***Median (IQR)*** |
| --- | --- | --- | --- |
| S1 | S^VV^S^VT^ | 1.05 (±1.614) | 1 (0~2) |
|  | A^VV^S^VT^ | 0.938 (±1.641) | 1 (0~2) |
|  | S^VV^A^VT^ | 0.462 (±1.896) | 1 (-1~2) |
|  | A^VV^A^VT^ | 0.062 (±1.97) | 1 (-2~2) |
| S2 | S^VV^S^VT^ | 1.862 (±1.156) | 2 (1~3) |
|  | A^VV^S^VT^ | 1.175 (±1.784) | 2 (0~3) |
|  | S^VV^A^VT^ | 0.613 (±2.047) | 1 (-1~2) |
|  | A^VV^A^VT^ | -0.225 (±1.936) | 0 (-2~1) |
| S3 | S^VV^S^VT^ | 2.212 (±0.867) | 2 (2~3) |
|  | A^VV^S^VT^ | 2.075 (±1.041) | 2 (2~3) |
|  | S^VV^A^VT^ | 0.338 (±2.105) | 1 (-1.25~2.25) |
|  | A^VV^A^VT^ | 0.125 (±2.218) | 0 (-2~2) |
| S4 | S^VV^S^VT^ | -1.038 (±1.724) | -2 (-2~0) |
|  | A^VV^S^VT^ | -0.787 (±1.791) | -1 (-2~0.25) |
|  | S^VV^A^VT^ | -0.9 (±1.797) | -1 (-2~0) |
|  | A^VV^A^VT^ | -0.725 (±1.896) | -1 (-2~1) |
| S5 | S^VV^S^VT^ | -1.138 (±1.674) | -2 (-2~0) |
|  | A^VV^S^VT^ | -0.925 (±1.712) | -1 (-2~0.25) |
|  | S^VV^A^VT^ | -1.312 (±1.588) | -2 (-3~0) |
|  | A^VV^A^VT^ | -1.337 (±1.645) | -2 (-3~0) |
| S6 | S^VV^S^VT^ | 0.2 (±1.845) | 1 (-2~1) |
|  | A^VV^S^VT^ | -0.025 (±1.842) | 1 (-2~1) |
|  | S^VV^A^VT^ | 0.088 (±1.794) | 1 (-2~1) |
|  | A^VV^A^VT^ | -0.013 (±1.79) | 1 (-2~1) |
| S7 | S^VV^S^VT^ | 6.475 (±2.077) | 7 (5~8) |
|  | A^VV^S^VT^ | 5.763 (±2.038) | 6 (4~7) |
|  | S^VV^A^VT^ | 5.825 (±2.209) | 6 (4~8) |
|  | A^VV^A^VT^ | 5.312 (±2.32) | 6 (3~7) |

***S3 Table. Descriptive statistics for questionnaire results*** *(N = 80).*

**Post hoc paired comparisons for the questionnaire data**

| ***Question*** | ***Comparison*** | ***V*** | ***p value*** | ***Tails*** | ***BF_10_*** | ***Effect size (****r_C_****)*** |
| --- | --- | --- | --- | --- | --- | --- |
| S1 | S^VV^S^VT^ > S^VV^A^VT^ | 1217 | <0.001^***^ | 1 | 104.237 | 0.525 |
|  | S^VV^S^VT^ > A^VV^S^VT^ | 482 | 0.152 | 1 | 0.338 | 0.176 |
|  | S^VV^S^VT^ > A^VV^A^VT^ | 1227 | <0.001^***^ | 1 | 17289.84 | 0.715 |
|  | S^VV^A^VT^ ≠ A^VV^S^VT^ | 357 | 0.005^**^ | 2 | 6.124 | -0.44 |
|  | S^VV^A^VT^ > A^VV^A^VT^ | 760 | 0.007^**^ | 1 | 5.708 | 0.406 |
|  | A^VV^S^VT^ > A^VV^A^VT^ | 1094 | <0.001^***^ | 1 | 1459.099 | 0.65 |
| S2 | S^VV^S^VT^ > S^VV^A^VT^ | 1125 | <0.001^***^ | 1 | 74329.31 | 0.765 |
|  | S^VV^S^VT^ > A^VV^S^VT^ | 899.5 | 0.002^**^ | 1 | 30.803 | 0.469 |
|  | S^VV^S^VT^ > A^VV^A^VT^ | 2022.5 | <0.001^***^ | 1 | 1.24E+10 | 0.886 |
|  | S^VV^A^VT^ ≠ A^VV^S^VT^ | 724.5 | 0.076 | 2 | 0.615 | -0.258 |
|  | S^VV^A^VT^ > A^VV^A^VT^ | 1252.5 | 0.001^**^ | 1 | 33.057 | 0.464 |
|  | A^VV^S^VT^ > A^VV^A^VT^ | 1633 | <0.001^***^ | 1 | 183532.3 | 0.727 |
| S3 | S^VV^S^VT^ > S^VV^A^VT^ | 1464 | <0.001^***^ | 1 | 8.55E+10 | 0.972 |
|  | S^VV^S^VT^ > A^VV^S^VT^ | 428 | 0.108 | 1 | 0.551 | 0.218 |
|  | S^VV^S^VT^ > A^VV^A^VT^ | 1730 | <0.001^***^ | 1 | 1.2E+11 | 0.955 |
|  | S^VV^A^VT^ ≠ A^VV^S^VT^ | 73.5 | <0.001^***^ | 2 | 9.26E+08 | -0.914 |
|  | S^VV^A^VT^ > A^VV^A^VT^ | 554.5 | 0.161 | 1 | 0.377 | 0.172 |
|  | A^VV^S^VT^ > A^VV^A^VT^ | 1829 | <0.001^***^ | 1 | 1.98E+09 | 0.873 |
| S4 | S^VV^S^VT^ > S^VV^A^VT^ | 523 | 0.754 | 1 | 0.075 | -0.111 |
|  | S^VV^S^VT^ > A^VV^S^VT^ | 261 | 0.95 | 1 | 0.048 | -0.296 |
|  | S^VV^S^VT^ > A^VV^A^VT^ | 459 | 0.941 | 1 | 0.049 | -0.251 |
|  | S^VV^A^VT^ ≠ A^VV^S^VT^ | 537 | 0.443 | 2 | 0.15 | -0.123 |
|  | S^VV^A^VT^ > A^VV^A^VT^ | 289 | 0.956 | 1 | 0.056 | -0.295 |
|  | A^VV^S^VT^ > A^VV^A^VT^ | 523 | 0.579 | 1 | 0.095 | -0.032 |
| S5 | S^VV^S^VT^ > S^VV^A^VT^ | 413.5 | 0.099 | 1 | 0.57 | 0.242 |
|  | S^VV^S^VT^ > A^VV^S^VT^ | 130.5 | 0.975 | 1 | 0.043 | -0.4 |
|  | S^VV^S^VT^ > A^VV^A^VT^ | 478.5 | 0.101 | 1 | 0.64 | 0.227 |
|  | S^VV^A^VT^ ≠ A^VV^S^VT^ | 172 | 0.005^**^ | 2 | 11.75 | -0.511 |
|  | S^VV^A^VT^ > A^VV^A^VT^ | 329 | 0.529 | 1 | 0.143 | -0.012 |
|  | A^VV^S^VT^ > A^VV^A^VT^ | 455.5 | 0.003^**^ | 1 | 12.686 | 0.531 |
| S6 | S^VV^S^VT^ > S^VV^A^VT^ | 440.5 | 0.141 | 1 | 0.379 | 0.189 |
|  | S^VV^S^VT^ > A^VV^S^VT^ | 488 | 0.081 | 1 | 0.587 | 0.251 |
|  | S^VV^S^VT^ > A^VV^A^VT^ | 504.5 | 0.098 | 1 | 0.593 | 0.23 |
|  | S^VV^A^VT^ ≠ A^VV^S^VT^ | 472.5 | 0.578 | 2 | 0.159 | 0.098 |
|  | S^VV^A^VT^ > A^VV^A^VT^ | 445 | 0.316 | 1 | 0.23 | 0.085 |
|  | A^VV^S^VT^ > A^VV^A^VT^ | 430.5 | 0.503 | 1 | 0.116 | 0 |
| S7 | S^VV^S^VT^ > S^VV^A^VT^ | 1315.5 | <0.001^***^ | 1 | 91.613 | 0.486 |
|  | S^VV^S^VT^ > A^VV^S^VT^ | 1174.5 | 0.001^**^ | 1 | 41.136 | 0.472 |
|  | S^VV^S^VT^ > A^VV^A^VT^ | 1641.5 | <0.001^***^ | 1 | 31463.8 | 0.681 |
|  | S^VV^A^VT^ ≠ A^VV^S^VT^ | 833.5 | 0.958 | 2 | 0.127 | 0.008 |
|  | S^VV^A^VT^ > A^VV^A^VT^ | 939 | 0.005^**^ | 1 | 4.808 | 0.416 |
|  | A^VV^S^VT^ > A^VV^A^VT^ | 1174.5 | 0.013^*^ | 1 | 3.573 | 0.327 |

***S4 Table. Quantitative statistics for the questionnaire results*** *(N = 80). The Wilcoxon signed-rank test was used. We tested the one-sided hypotheses that S^VV^S^VT^>A^VV^S^VT^, S^VV^S^VT^> S^VV^A^VT^, S^VV^S^VT^>A^VV^A^VT^, A^VV^S^VT^> A^VV^A^VT^ and S^VV^A^VT^> A^VV^A^VT^ and the two-sided hypothesis that A^VV^S^VT^≠ S^VV^A^VT^. “V” shows the sum of positive ranks, “p” the p value, “BF_10_” the Bayesian factor for the alternative hypothesis, and “r_C_” the matched pairs rank biserial correlation as the effect size.*

*Note: *** < 0.001, **< 0.01, *< 0.05*

Section III – SCR Extra analysis

In our study, we could see a raw value <.01 in two subjects; therefore, below, we report three extra analyses. We analyzed the data of all subjects without normalization (extra analysis 1). Then, we excluded two extra subjects whose SCR response was <.01 (in Subject 1, 37.5% and in Subject 2, 62.5% of responses were 0). The total of their null responses is 0.02% of the entire sample SCR responses. We therefore performed the same robust linear mixed model and the same post hoc comparisons as in the main analysis (therefore with normalized data; see main manuscript for data analysis details) in the new sample (N =48) (extra analysis 2), and then we normalized the magnitude of the new sample (N = 48) and ran the same analysis (extra analysis 3). We refer to the normalization with Z scores as Z = value/highest response.

*First extra analysis:*


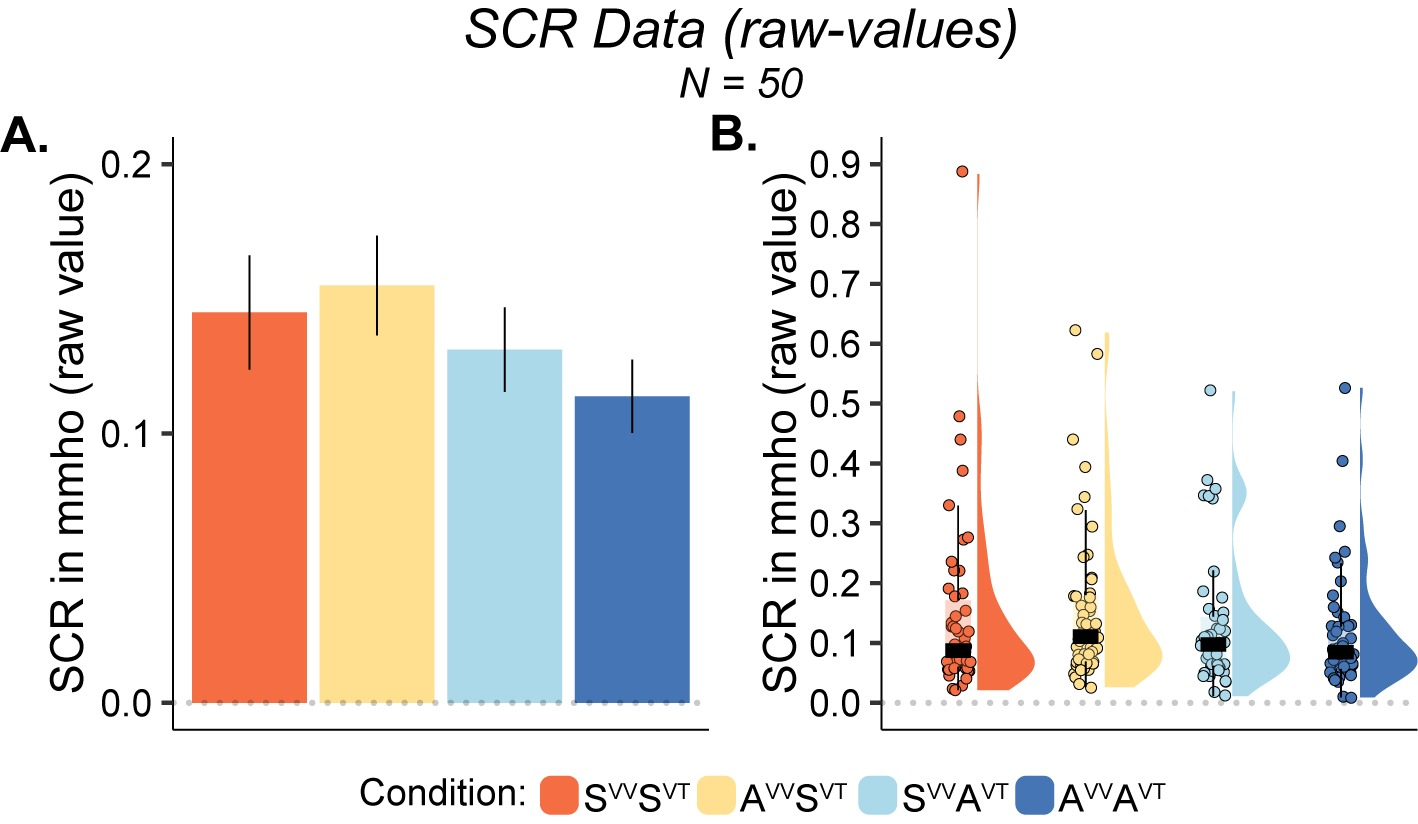


***S4 Fig. Non-normalized SCR data (N = 50).*** *(A) Barplot for SCR data (raw values). (B) shows the distribution, individual data points and medians of SCR data (raw values).*

| *Name* | *Estimate* | *t value* | *p value* |
| --- | --- | --- | --- |
| Intercept | 0.095 | 9.472 | 0 |
| Sequence | -0.001 | -1.568 | 0.117 |
| Visuo-Tactile-Synch | 0.021 | 3.41 | 0.001^**^ |
| Visuo-Vestibular-Synch | 0.007 | 1.131 | 0.258 |
| Visuo-Tactile-Synch*Visuo-Vestibular-Synch | -0.012 | -1.335 | 0.182 |

***S5 Table. Quantitative statistics for the SCR nonnormalized results*** *(N = 50). A robust linear mixed model (rlmer) was run: Value ~ sequence + visuo_tactile*visuo_vestibular + (1|Participant).*

*Note: *** < 0.001, **< 0.01, *< 0.05*

| *Comparison* | *V* | *p* | *BF_10_* | *r_C_* | *tails* |
| --- | --- | --- | --- | --- | --- |
| S^VV^A^VT^ > A^VV^A^VT^ | 819 | 0.04^*^ | 0.725 | 0.285 | greater |
| A^VV^S^VT^ > A^VV^A^VT^ | 1023 | 0^***^ | 7.437 | 0.605 | greater |
| S^VV^S^VT^ > A^VV^A^VT^ | 900 | 0.002^**^ | 1.255 | 0.469 | greater |
| S^VV^S^VT^ > A^VV^S^VT^ | 472.5 | 0.945 | 0.081 | -0.259 | greater |
| S^VV^S^VT^ > S^VV^A^VT^ | 737 | 0.171 | 0.648 | 0.156 | greater |
| S^VV^A^VT^ ≠ A^VV^S^VT^ | 364 | 0.008^**^ | 2.444 | -0.429 | two sided |

***S6 Table. Quantitative statistics for the SCR nonnormalized results*** *(N = 50). The Wilcoxon signed-rank test was used. We tested the one-sided hypotheses that S^VV^A^VT^> A^VV^A^VT^, A^VV^S^VT^> A^VV^A^VT^, S^VV^S^VT^>A^VV^A^VT^, S^VV^S^VT^>A^VV^S^VT^, S^VV^S^VT^> S^VV^A^VT^ and the two-sided hypothesis that A^VV^S^VT^≠ S^VV^A^VT^. “V” shows the sum of positive ranks, “p” the p value, “BF_10_” the Bayesian factor for the alternative hypothesis, and “r_C_” the matched pairs rank biserial correlation as the effect size.*

*Note: *** < 0.001, **< 0.01, *< 0.05*

*Second extra analysis:*


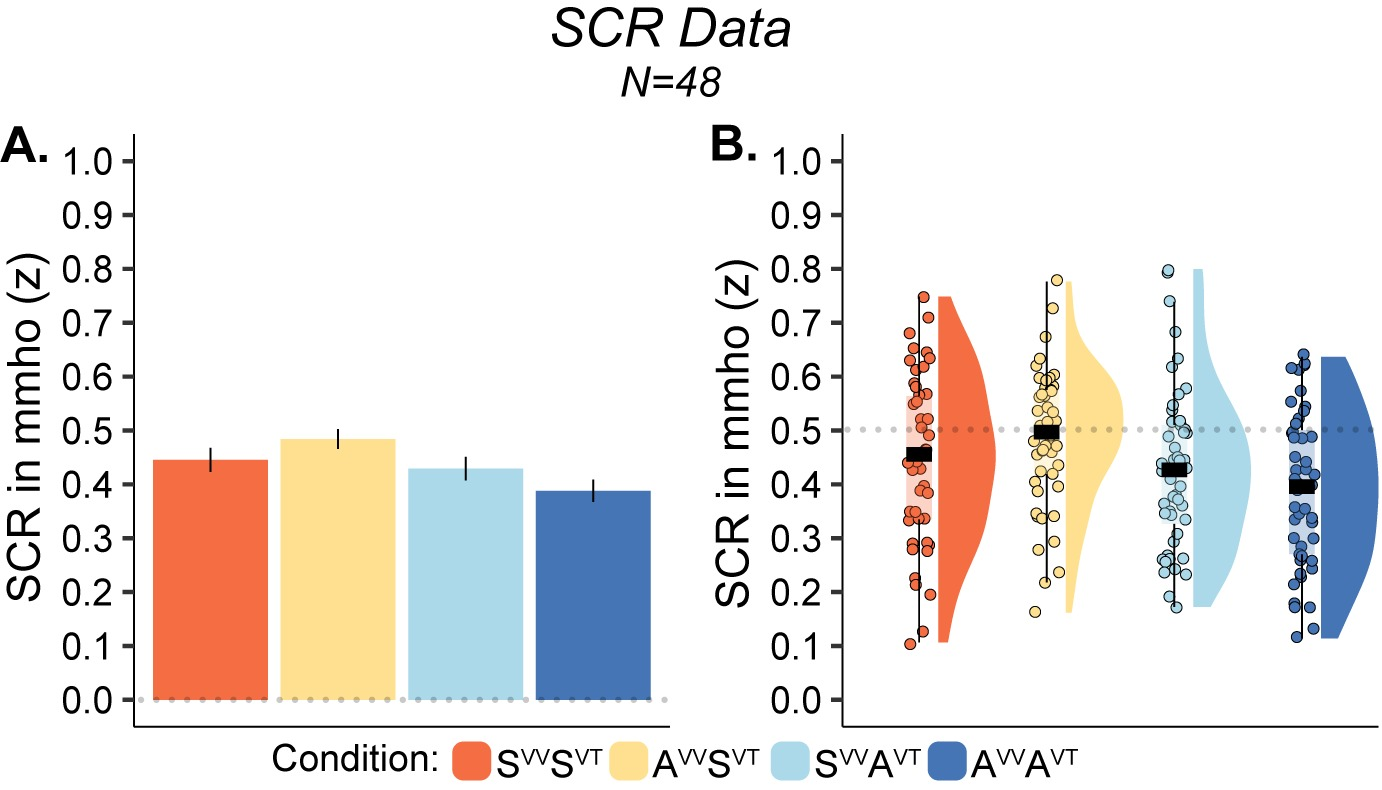


***S5 Fig. Normalized-SCR data (N = 48).*** *(A) Bar plot for normalized SCR data. (B) shows the distribution, individual data points and medians of normalized SCR data.*

| *Name* | *Estimate* | *t value* | *p value* |
| --- | --- | --- | --- |
| Intercept | 0.4 | 13.775 | 0 |
| Sequence | -0.003 | -1.822 | 0.069 |
| Visuo-Tactile-Synch | 0.086 | 3.437 | 0.001^**^ |
| Visuo-Vestibular-Synch | 0.032 | 1.293 | 0.196 |
| Visuo-Tactile-Synch*Visuo-Vestibular-Synch | -0.063 | -1.784 | 0.074 |

***S7 Table. Quantitative statistics for the normalized SCR results*** *(N = 48). A robust linear mixed model (rlmer) was run: Value ~ sequence + visuo_tactile*visuo_vestibular + (1|Participant).*

*Note: *** < 0.001, **< 0.01, *< 0.05*

| *Comparison* | *V* | *p* | *BF_10_* | *d_z_* | *tails* |
| --- | --- | --- | --- | --- | --- |
| S^VV^A^VT^ > A^VV^A^VT^ | 1.885 | 0.033* | 1.54 | 0.272 | greater |
| A^VV^S^VT^ > A^VV^A^VT^ | 4.163 | 0*** | 358.053 | 0.601 | greater |
| S^VV^S^VT^ > A^VV^A^VT^ | 2.283 | 0.014* | 3.239 | 0.329 | greater |
| S^VV^S^VT^ > A^VV^S^VT^ | -1.903 | 0.968 | 0.058 | -0.275 | greater |
| S^VV^S^VT^ > S^VV^A^VT^ | 0.663 | 0.255 | 0.285 | 0.096 | greater |
| S^VV^A^VT^ ≠ A^VV^S^VT^ | -2.347 | 0.023* | 1.869 | -0.339 | two sided |

***S8 Table. Quantitative statistics for the normalized SCR results*** *(N = 48). The Wilcoxon signed-rank test was used. We tested the one-sided hypotheses that S^VV^A^VT^> A^VV^A^VT^, A^VV^S^VT^> A^VV^A^VT^, S^VV^S^VT^>A^VV^A^VT^, S^VV^S^VT^>A^VV^S^VT^, S^VV^S^VT^> S^VV^A^VT^ and the two-sided hypothesis that A^VV^S^VT^≠ S^VV^A^VT^. “V” shows the sum of positive ranks, “p” the p value, “BF_10_” the Bayesian factor for the alternative hypothesis, and “r_C_” the matched pairs rank biserial correlation as the effect size.*

*Note: *** < 0.001, **< 0.01, *< 0.05*

*Third extra analysis:*


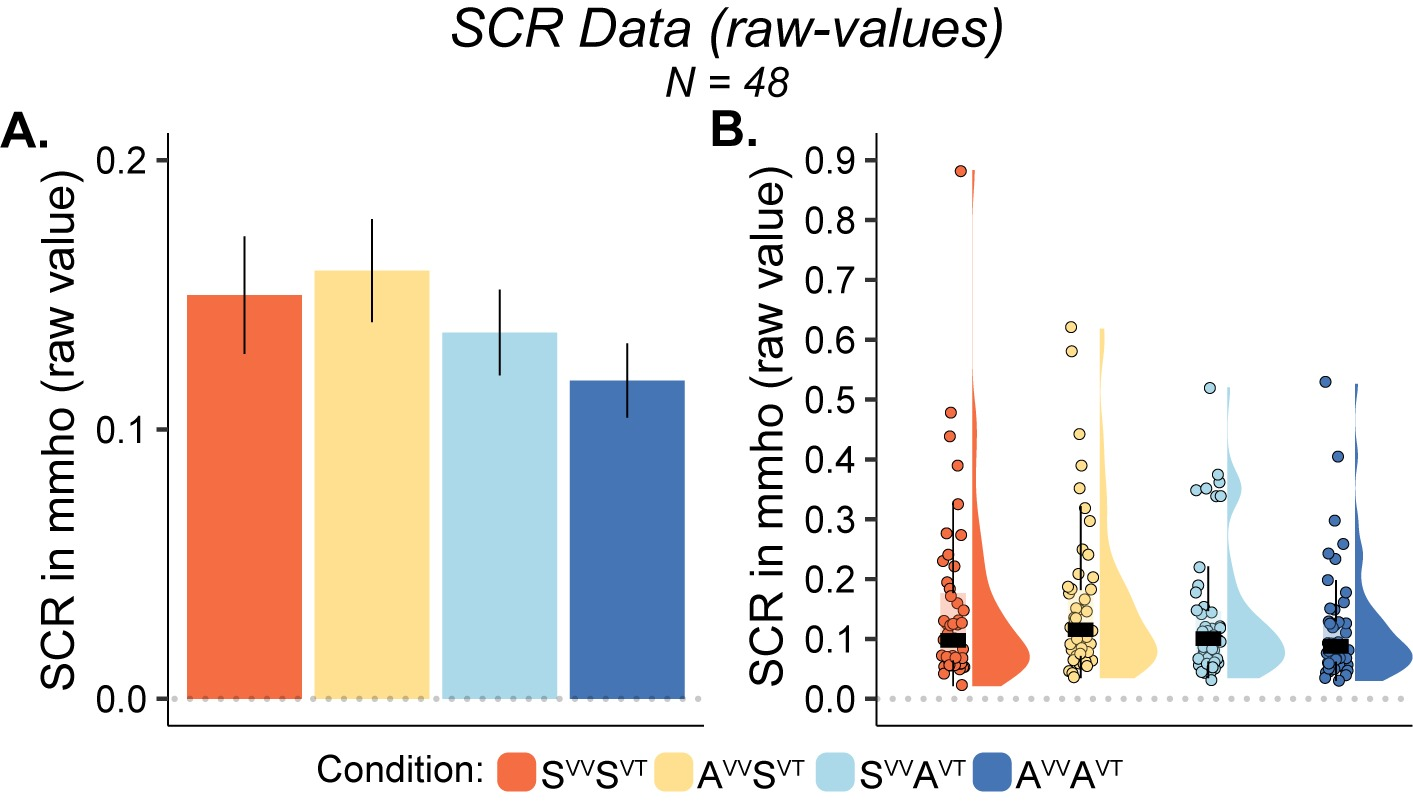


***S6 Fig. Non-normalized SCR data (N = 48).*** *(A) Barplot for SCR data (raw values). (B) shows the distribution, individual data points and medians of SCR data (raw values).*

| *Name* | *Estimate* | *t value* | *p value* |
| --- | --- | --- | --- |
| Intercept | 0.099 | 9.586 | 0 |
| Sequence | -0.001 | -1.408 | 0.159 |
| Visuo-Tactile-Synch | 0.021 | 3.136 | 0.002^**^ |
| Visuo-Vestibular-Synch | 0.007 | 1.107 | 0.268 |
| Visuo-Tactile-Synch*Visuo-Vestibular-Synch | -0.011 | -1.221 | 0.222 |

***S9 Table. Quantitative statistics for the SCR nonnormalized results*** *(N = 48). A robust linear mixed model (rlmer) was run: Value ~ sequence + visuo_tactile*visuo_vestibular + (1|Participant).*

*Note: *** < 0.001, **< 0.01, *< 0.05*

| *Comparison* | *V* | *p* | *BF_10_* | *r_C_* | *tails* |
| --- | --- | --- | --- | --- | --- |
| S^VV^A^VT^ > A^VV^A^VT^ | 754 | 0.045^*^ | 0.718 | 0.282 | greater |
| A^VV^S^VT^ > A^VV^A^VT^ | 933 | 0^***^ | 5.71 | 0.587 | greater |
| S^VV^S^VT^ > A^VV^A^VT^ | 818 | 0.004^**^ | 1.189 | 0.45 | greater |
| S^VV^S^VT^ > A^VV^S^VT^ | 445.5 | 0.929 | 0.088 | -0.242 | greater |
| S^VV^S^VT^ > S^VV^A^VT^ | 671 | 0.2 | 0.615 | 0.141 | greater |
| S^VV^A^VT^ ≠ A^VV^S^VT^ | 348 | 0.013^*^ | 1.763 | -0.408 | two sided |

***S10 Table. Quantitative statistics for the SCR nonnormalized results*** *(N = 48). The Wilcoxon signed-rank test was used. We tested the one-sided hypotheses that S^VV^A^VT^> A^VV^A^VT^, A^VV^S^VT^> A^VV^A^VT^, S^VV^S^VT^>A^VV^A^VT^, S^VV^S^VT^>A^VV^S^VT^, S^VV^S^VT^> S^VV^A^VT^ and the two-sided hypothesis that A^VV^S^VT^≠ S^VV^A^VT^. “V” shows the sum of positive ranks, “p” the p value, “BF_10_” the Bayesian factor for the alternative hypothesis, and “r_C_” the matched pairs rank biserial correlation as the effect size.*

*Note: *** < 0.001, **< 0.01, *< 0.05*

**References**

1. Oldfield RC. The assessment and analysis of handedness: The Edinburgh inventory. Neuropsychologia. 1971;9:97–113.

https://doi.org/10.1016/0028-3932(71)90067-4

2. Petkova VI, Ehrsson HH. If I Were You: Perceptual Illusion of Body Swapping. PLOS ONE 2008;3:e3832.

https://doi.org/10.1371/journal.pone.0003832

3. Kerby DS. The simple difference formula: An approach to teaching nonparametric correlation. Compr Psychol. 2014;3:11. IT. 3.1.

https://doi.org/10.2466/11.IT.3.1

4. King BM, Rosopa PJ, Minium EW. Statistical reasoning in the behavioral sciences. John Wiley & Sons; 2018.

5. Champely S, Ekstrom C, Dalgaard P, Gill J, Weibelzahl S, Anandkumar A, Ford C, Volcic R, De Rosario H, De Rosario MH. Package ‘pwr’. R Package Version. 2018;1(2).

6. Cohen J. Quantitative methods in psychology: A power primer. In: Psychological bulletin. Citeseer; 1992.
